# Diverse novel *Wolbachia *bacteria strains and widespread co-infections with *Asaia *bacteria in Culicine mosquitoes from ecologically diverse regions of Cameroon

**DOI:** 10.12688/wellcomeopenres.18580.1

**Published:** 2023-06-21

**Authors:** Aina Mercant Osuna, Alexandra Gidley, Marie Paul Audrey Mayi, Roland Bamou, Vishaal Dhokiya, Christophe Antonio-Nkondjio, Claire Louise Jeffries, Thomas Walker

**Affiliations:** 1 Department of Disease Control, London School of Hygiene & Tropical Medicine, London, UK; 2 Vector Borne Diseases Laboratory of the Research Unit of Biology and Applied Ecology (VBID-RUBAE), Department of Animal Biology, University of Dschang, Dschang, Cameroon; 3 Laboratory of Malaria and Vector Research, NIAID, NIH, Rockville, Maryland, USA; 4 IHU Méditerranée Infection, Marseille, France; 5 Vecteurs-Infections Tropicales et Méditerranéennes (VITROME), Aix Marseille University, Marseille, France; 6 Organisation de Coordination pour la lutte Contre les Endémies en Afrique Centrale (OCEAC), Yaoundé, Cameroon; 7 Department of Vector Biology, Liverpool School of Tropical Medicine, Liverpool, UK; 8 School of Life Sciences, University of Warwick, Coventry, UK

**Keywords:** Wolbachia, bacteria, mosquitoes

## Abstract

**Background: **The endosymbiotic bacterium *Wolbachia *infects numerous species of insects and *Wolbachia *transinfection of *Aedes* mosquito species is now being used for biocontrol programs as *Wolbachia *strains can both inhibit arboviruses and invade wild mosquito populations. The discovery of novel, resident *Wolbachia* strains in mosquito species warrants further investigation as potential candidate strains for biocontrol strategies.

**Methods: **We obtained mosquito specimens from diverse Culicine mosquitoes from Cameroon including ecologically diverse locations in the Central and West Regions. *Wolbachia* prevalence rates were assessed in addition to the environmentally acquired bacterial species *Asaia *in major Culicine genera. PCR-based methods were also used with phylogenetic analysis to confirm identities of host mosquito species and *Wolbachia* strains were classified using multi-locus sequence typing (MLST).

**Results**: We report high *Wolbachia* prevalence rates for Culicine species, including in a large cohort of *Aedes africanus* collected from west Cameroon in which 100% of mono-specific pools were infected. Furthermore, co-infections with *Asaia* bacteria were observed across multiple genera, demonstrating that these two bacteria can co-exist in wild mosquito populations. *Wolbachia* strain MLST and phylogenetic analysis provided evidence for diverse *Wolbachia *strains in 13 different mosquito species across seven different genera.  Full or partial MLST profiles were generated from resident *Wolbachia* strains in six *Culex* species ( *quinquefasciatus*, *watti*, *cinerus, nigripalpus, perexiguus *and *rima), *two *Aedes *species *(africanus *and *denderensis) *and in *Mansonia uniformis, Catageiomyia argenteopunctata, Lutzia tigripes, Eretmapodites chrysogaster *and *Uranotaenia bilineata. *

**Conclusions:** Our study provides further evidence that *Wolbachia* is widespread within wild mosquito populations of diverse Culicine species and provides further candidate strains that could be investigated as future options for *Wolbachia*-based biocontrol to inhibit arbovirus transmission.

## Introduction

*Wolbachia* are endosymbiotic bacteria which reside within an estimated 40–70% of insect species ^1, 2^. These bacteria have been detected in numerous mosquitoes that transmit human pathogens including species within the *Aedes* ( *Ae*.), *Culex* ( *Cx*.) and *Anopheles* ( *An*.) genera ^3– 9^. *Wolbachia* is now being used in applied mosquito biocontrol strategies as strains inhibit arboviruses and invade mosquito populations using the reproductive phenotype cytoplasmic incompatibility (CI). *Wolbachia*-induced CI was first used in mosquito release control trials in the 1960s in attempts to supress *Cx. quinquefasciatus* populations in Myanmar ^10^. More recently, *Wolbachia*-infected *Aedes* males have been released to induce CI and the associated sterility from matings with wild-type females, resulting in inviable progeny. This method of suppressing the population, the incompatible insect technique (IIT), has seen field trials for species such as *Ae. albopictus* that contain natural resident strains ^11, 12^. Embryo microinjection has also resulted in transinfected strains in *Ae. aegypti* ^13– 16^. These transinfected strains, including *w*Mel, have been shown to invade wild mosquito populations and also inhibit major arbovirus transmission, such as dengue virus (DENV) ^13, 15, 17– 19^. The *w*Mel *Ae. aegypti* line, through release programmes, is now present in more than 10 countries and encouragingly, a randomised control trial in Indonesia resulted in a 77% DENV inhibition ^20^. The *w*Mel strain is being continually released into additional DENV endemic countries, and based on mathematical modelling, has the capacity to reduce the DENV R0 (basic reproduction number) from 66–75% ^21^. The *w*Mel strain also inhibits other medically important arboviruses such as chikungunya virus (CHIKV) ^22^, Yellow fever virus (YFV) ^23^ and Zika virus (ZIKV) ^24^. 

*Wolbachia* strains that naturally reside within mosquito populations can provide important comparative data to complement biocontrol strategies. For example, whether *Wolbachia* strains in natural populations are found at a high prevalence, and whether strains are capable of inducing CI to allow rapid population invasion. The presence of other bacteria, such as *Asaia,* has been postulated to compete with *Wolbachia* to colonise the reproductive tissues of mosquitoes, including the ovaries in females ^25, 26^. Therefore, examining the possibility of co-infections in diverse mosquito populations will investigate if any antagonistic associations between these two common bacteria are present, as has been demonstrated in lab populations ^25, 26^. Numerous studies which have detected natural *Wolbachia* strains in Culicines have undertaken analysis of the *16S rRNA* gene when looking at the wider microbiome ^27, 28^. However, a more targeted approach amplifying *Wolbachia*-specific genes is required to confirm a resident strain is present and phylogenetic analysis allows any newly discovered strains to be compared to existing strains. *Wolbachia* strains can be classified in Supergroups designated from A to H. A and B Supergroup strains are mostly found in arthropods, with only one strain per host, or multiple strains infecting the same host ^29^. Superinfection, the infection of more than one strain of *Wolbachia* in the same host – as can be seen in *Ae. albopictus* with the *w*AlbA and *w*AlbB strains ^30^ – can comprise strains of the same Supergroup, or differing Supergroups, such as A and B group strain co-infections.

Cameroon is a country in West Africa in which outbreaks of arboviral diseases including DENV, YFV, CHIKV and Rift Valley fever virus (RVFV) ^31, 32^ are a possibility. Cases of arboviruses are reported throughout the country and there is concern that rapid urbanisation seen throughout Africa could exacerbate transmission through favouring the breeding conditions of urban-adapted mosquito species ^33^. Deforestation has a significant effect on the abundance and diversity of Cameroon mosquito species and could lead to spill-over transmission of additional circulating zoonotic viruses such as Semliki Forest, o’nyong’nyong and Bwamba viruses ^34^. Here, we obtained specimens from entomological surveys undertaken in Central and West Cameroon to determine if diverse Culicine species harbour resident *Wolbachia* strains and if co-infections exist with *Asaia*, given this bacterium can be environmentally acquired and can compete with *Wolbachia*. A combination of phylogenetic analysis and strain typing using multi-locus sequence typing (MLST) revealed a diversity of newly discovered *Wolbachia* strains. 

## Methods

### Mosquito collections

**Figure 1.**
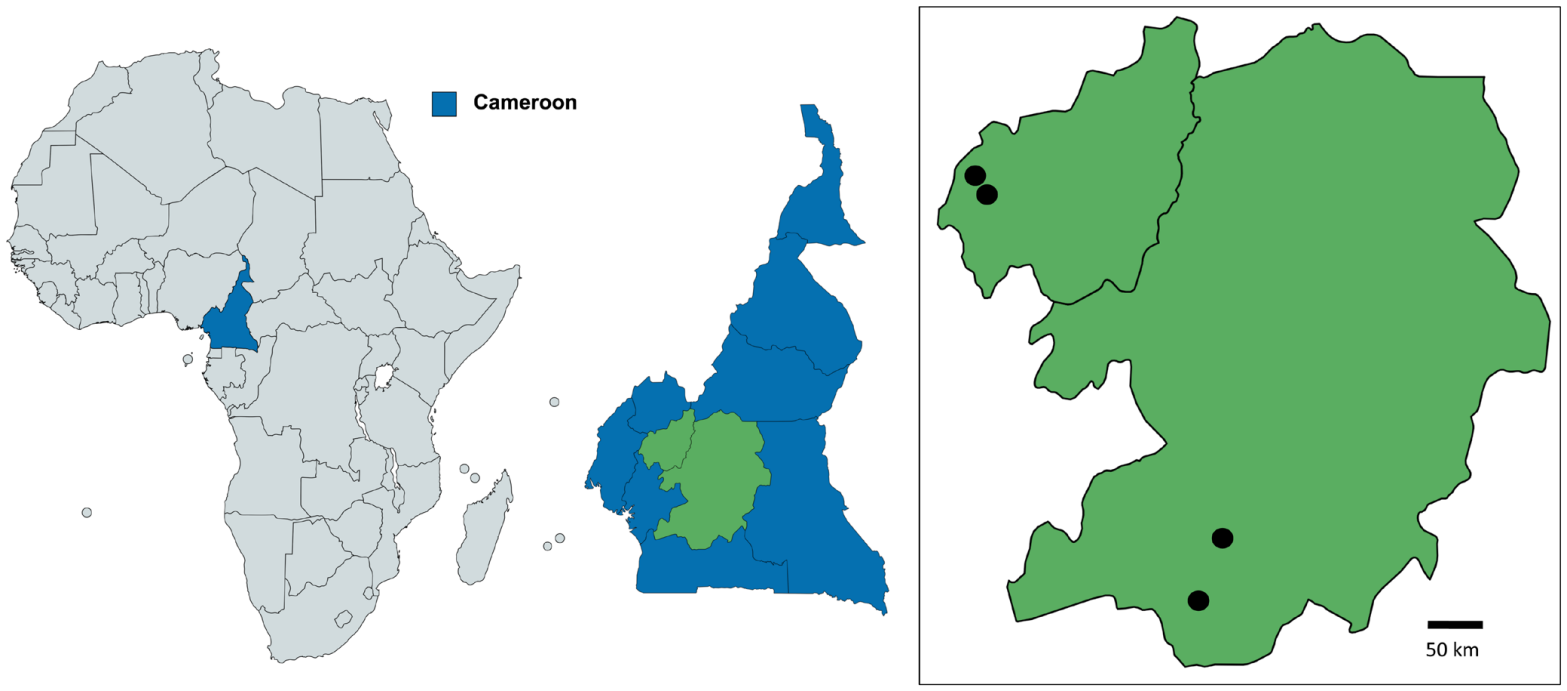
Mosquito collection sites in Cameroon. Collection sites in the Western Province/region were Fonakeukeu (05°24′73′′ N, 010°04′79′′ E) and Toutsang (05°25′65′′ N, 010°04′05′′ E). Collection sites in the Central Province/regions included Yaoundé (3°52'22.2"N 11°30'38.0"E) and Olama village (3°24'45.0"N 11°17'03.0"E). Maps were produced using Mapchart licensed under a Creative Commons Attribution-ShareAlike 4.0 International License.

A variety of adult mosquito trapping methods were used in the Central Region of Cameroon in Yaoundé (3°52’22.2”N, 11°30’38.0”E) and Olama village (3°24’45.0”N 11°17’03.0”E) from June 2010 to July 2019. Yaoundé (the capital of Cameroon) is an urban location characterised by mosquito breeding sites such as tyres, containers, and temporary water pools. In contrast, Olama village is rural, located along the Nyong River ( Figure 1) and is characterised by houses built with corrugated metal roofs and mud walls. In Olama village, eight CDC miniature light traps were hung in four houses and two Stealth traps (model 14 which uses ultraviolet light for attraction) were hung in one house (one trap inside and one outside). BG Suna traps containing a carbon dioxide source from fermented yeast as an attractant were hung in trees nearby houses, approximately one metre above the ground. Traps were powered using a 12V battery over a period of 11 days from 17:00–07:00. Human landing catches were carried out in Olama Village as previously described ^4^. In Yaoundé, BG Sentinel-2 traps containing a BG Sweetscent lure used as an attractant were used to collect mosquitoes for 15 days from 16:30–10:00. Traps were assembled using manufacturers guidelines and positioned nearby potential *Aedes* breeding sites under tree coverage. Larval collections using ladles and sieves were also carried out in Yaoundé́ in typical urban breeding sites from the following districts: Etoude, Nkolbissim and Briqueterie.

Mosquitoes were collected in the West Region from the Menoua division as previously described ^35^ and from two locations in the Dschang sub-division in a rural area; Fonakeukeu (05°24′73′′ N, 010°04′79′′ E) and a peri-urban area; Toutsang (05°25′65′′ N, 010°04′05′′ E). Collections were carried out in both the rainy season (from March to September 2019) and dry season (from November 2019 to February 2020) using sweep nets. Mosquitoes from Central Cameroon were morphologically identified to genera level, and from West Cameroon to species level, using morphological identification keys, then preserved in RNAlater (Invitrogen) and kept at below -20°C, prior to being sent for molecular analysis at LSHTM. 

### RNA extraction and generation of complimentary DNA (cDNA)

From West Cameroon collections, mosquitoes of the same species, from the same location and season, were pooled prior to RNA extraction (650 mosquitoes, 192 pools). From Central Cameroon collections, a sub-sample (n=576) was selected for molecular analysis based on diversity of genera and collection sites. Mosquito RNA was extracted from either pools or individuals using Qiagen 96 RNeasy Kits and a Qiagen Tissue Lyser II (Hilden, Germany) with 5 mm stainless steel beads (Qiagen) to homogenize mosquitoes. Resulting RNA was eluted in 45 µL of RNase-free water and stored at −70°C. cDNA was generated from RNA using an Applied Biosystems High-Capacity cDNA Reverse Transcription kit. Each reaction (20 µL) was made up of the following: 1 µL reverse transcriptase, 2 µL 10X RT buffer, 2 µL 10X random primers, 0.8 µL 25X dNTP (100 mM), 4.2 µL nuclease-free water and 10 µL RNA. A Bio-Rad T100 Thermal Cycler was used to generate cDNA as follows: 25°C for 10 minutes (min), 37°C for 120 min; 85°C for 5 min with all resulting cDNA stored at –20°C.

### *Wolbachia* and *Asaia* detection

The detection of *Wolbachia* strains was initially carried out by amplification of three conserved *Wolbachia* genes; *16S rRNA* gene using primers W-Spec-16S-F: 5’-CATACCTATTCGAAGGGATA-3’ and W-Spec-16s-R: 5’-AGCTTCGAGTGAAACCAATTC-3’ ^36^, *Wolbachia* surface protein ( *wsp*) gene using primers wsp81F: 5’-TGGTCCAATAAGTGATGAAGAAAC-3’ and wsp691R: 5’-AAAAATTAAACGCTACTCCA-3’ ^37^, and *FtsZ* cell cycle gene using primers ftsZqPCR F: 5’-GCATTGCAGAGCTTGGACTT-3’ and ftsZqPCR R: 5’-TCTTCTCCTTCTGCCTCTCC-3’ ^2^. A Bio-Rad T100 Thermal Cycler using standard cycling conditions was used to amplify *16S rRNA* and *wsp* gene products which were then separated and visualised using an Invitrogen E-Gel iBase Real-Time Transilluminator with 2% SYBR safe E-Gel EX agarose gels. Real-time PCR reactions for the *ftsZ* and *16S rRNA* genes were carried out with 5 μL of FastStart SYBR Green Master mix (Roche Diagnostics), primers at a final concentration of 1 µM, 1 μL of PCR grade water and 2 μL cDNA (10 μL final volume/reaction). A Roche LightCycler 96 System was used to amplify PCR products using the following cycling conditions: 15 min at 95°C, 40 cycles of 95°C for 15 seconds (sec) and 58°C for 30 sec. *Asaia* detection was also carried out using Real time PCR by amplifying the 16S *rRNA* gene ^38^ with the same mastermix, reagent concentrations and cycling conditions as for *Wolbachia* genes. PCR assays included a dissociation curve (95°C for 10 sec, 65°C for 60 sec and 97°C for 1 sec) to check that the correct amplicon was being amplified. Fluorescence was quantified using LightCycler 96 software (Roche Diagnostics). 

### Molecular mosquito species identification

For *Aedes* mosquitoes collected from Central Cameroon sites, a SYBR-green based assay that can distinguish *Ae. aegypti* from *Ae. albopictus* based on the internal transcribed spacer 1 (ITS1) ^39^ was used. PCR cycling conditions for the ITS1 assay were: 95°C for 15 min, 40 cycles of 95°C for 10 sec, 55°C for 30 sec, 72°C for 20 sec and a dissociation curve (see above). For *Culex* mosquitoes collected from Central Cameroon sites, a multiplex PCR assay targeting the ACE1 gene ^40^ that can distinguish *Cx. pipiens pipiens* from *Cx. pipiens quinquefasciatus* was also undertaken, given these sibling species are morphologically indistinguishable *. PCR* cycling conditions for the ACE1: 95°C for 10 min, 34 cycles of 95°C for 30 sec, 55°C for 30 sec, 72°C for 1 min and 2°C for 5 min. To determine the species for additional samples that were *Wolbachia*-positive, Sanger sequencing and phylogenetic analysis of the cytochrome c oxidase subunit 1 ( *COI*) gene ^41^ was undertaken as this provided the most available sequences for comparison to ensure the optimal method for species identification.

### *Wolbachia* MLST

Five conserved genes ( *gatB*, *coxA*, *hcpA*, *ftsZ* and *fbpA)* were amplified to determine any newly discovered *Wolbachia* strains as previously described with the use of M13 adaptors or degenerate primers ^42^. MLST PCRs consisted of 10 µL of Phire Hot Start II PCR Master Mix (Thermo Scientific), primers with a final concentration of 1 µM, 1 µL of PCR-grade water and 2 µL template cDNA (20 µL total). PCR cycling was carried out in a Bio-Rad T100 Thermal Cycler using cycling conditions that were optimised for different MLST genes tested with the Phire Hot Start II PCR Master Mix. Three genes ( *gatB*, *hcpA* and *fbpA* genes) had the following cycling: 98°C for 30 sec, 34 cycles of 98°C for 5 sec, 65°C for 5 sec, 72°C for 10 sec, 72°C for 1 min. For two genes ( *coxA* and *ftsZ*) cycling was: 98°C for 30 sec, 34 cycles of 98°C for 5 sec, 55°C for 5 sec and 72°C for 30 sec, 72°C for 1 min.

### Sanger sequencing

PCR products considered worthy of sequencing, visualised using an Invitrogen E-Gel iBase Real-Time Transilluminator with 2% SYBR safe E-Gel EX agarose gels run for 10 mins, were selected and sent to Source BioScience (Nottingham, UK). PCR products were cleaned and forward and reverse sanger sequencing was undertaken by Source Bioscience. The MLST primers used were gene-specific and in the case of MLST genes just the M13 primers (M13_adaptor_F: 5’-TGTAAAACGACGGCCAGT-3’ and M13_adaptor_R: 5’-CAGGAAACAGCTATGACC-3’) were used if these adaptors were included in the initial PCR to generate the product. MEGAX ^43^ was used for all analysis of sequences with manual checking of both forward and reverse chromatograms. Editing of sequences included trimming and then alignment to produce consensus sequences was undertaken using ClustalW. Nucleotide BLAST (NCBI) database queries and searches against the *Wolbachia* MLST database were combined to determine if new alleles and strain types were present in our collection. We also submitted our sequences to GenBank and obtained accession numbers. 

### Phylogenetic analysis

Alignments were constructed in MEGAX and ClustalW was also used to align our sequences alongside additional sequences obtained from NCBI BLAST and *Wolbachia* MLST database searches. Maximum Likelihood (ML) phylogenetic trees were generated after initially determining the optimal nucleotide substitution model using the “Find Best-Fit Substitution Model (ML)” option within MEGAX. The lowest Bayesian Information Criterion (BIC) score was one of the criteria used and this resulted in two models: the Jukes-Cantor model ^44^ and the General Time Reversible model ^45^. For our phylogenetic analysis, we used the highest log likelihood and included next to the branches the percentage of trees in which the associated taxa clustered together. In all phylogenetic analyses we used a Bootstrap method with 1000 replications and Neighbor-Join and BioNJ algorithms using tMaximum Composite Likelihood (MCL). Our phylogenetic trees were then generated to scale, with branch lengths measured in the number of substitutions per site and we removed all gaps and missing data. 

### Ethical approval

We previously obtained permission and ethical approval for mosquito sampling ^4, 35^. Ethical approval for undertaking Human landing catches was obtained from the LSHTM ethics committee (reference no. 16684) in addition to local ethical approval (clearance no. 2016/01/685/CE/CNERSH/SP) delivered by the Cameroon National Ethics (CNE) Committee for Research on Human Health).

## Results

### *Wolbachia* and *Asaia* prevalence rates

**Figure 2.**
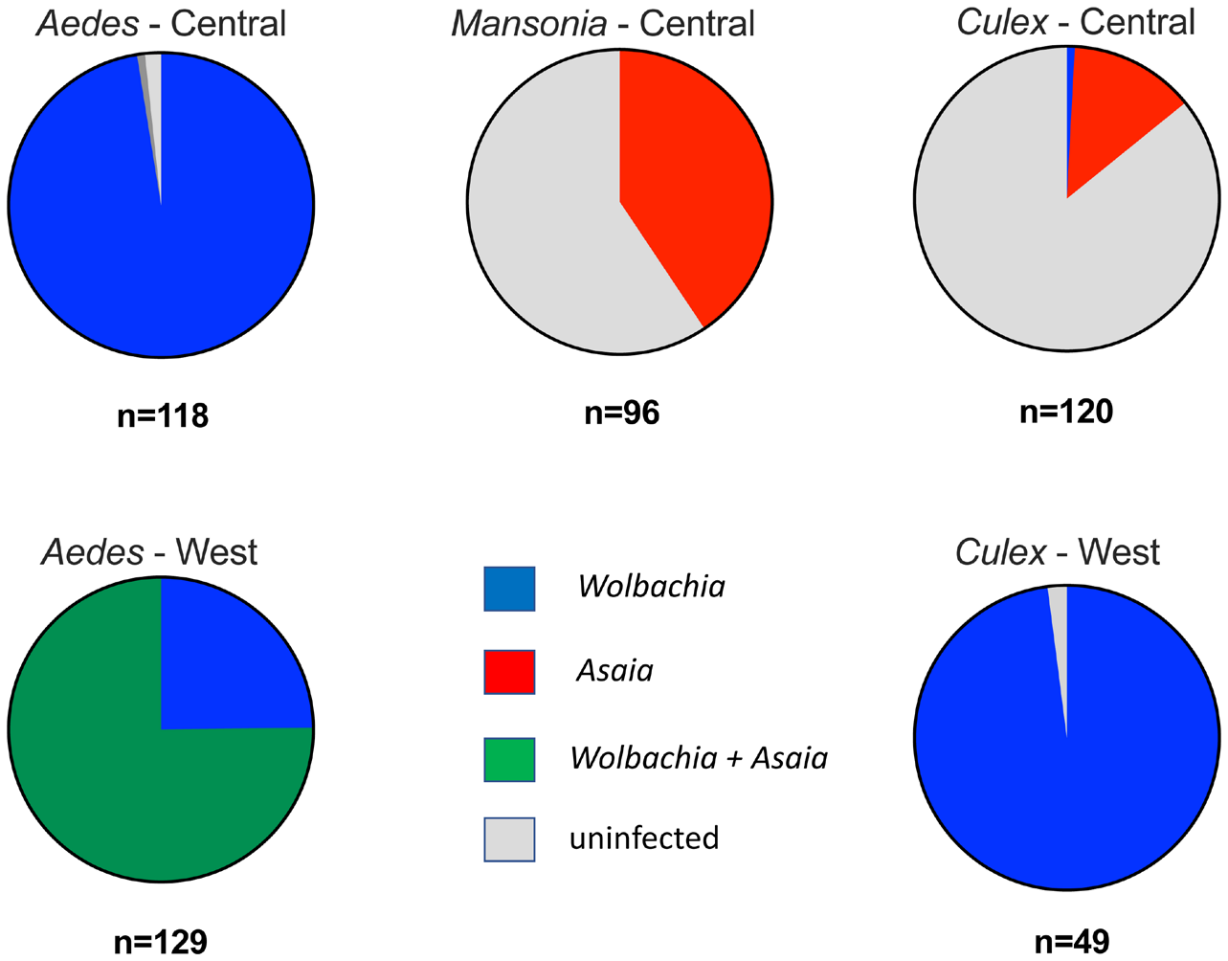
*Wolbachia*, *Asaia* and co-infection prevalence rates from major Culicine mosquito genera collected from the West and Central regions of Cameroon. Mosquitoes from the Central region were individually extracted and analysed. Mosquitoes from the West region were extracted from monospecific pools (same species from same collection location) and prevalence analysis represents pooled samples. No *Mansonia* mosquitoes were collected from the West region.

We compared the prevalence rates of *Wolbachia* and *Asaia* rates using the *16S rRNA gene* from the three major Culicine genera collected from both the West and Central Regions, with the caveat that the West Region samples were monospecific pools from the same species at the same location ( Figure 2, Table 1). In the Central Region, 97.96% (n=115) of *Aedes* genera mosquitoes were infected only with *Wolbachia*, and *Asaia* was only detected in a single mosquito as a coinfection. In contrast, the majority of *Culex* genera mosquitoes collected from the Central Region were uninfected (85.83%, 103), with *Asaia* detected in 13.45% (n=16) of individuals and only a single individual infected with *Wolbachia*. A similar infection prevalence was observed in *Mansonia* collected from the Central Region, but a higher prevalence of *Asaia* was detected (40.63%, n=39) and there was no *Wolbachia* detected (59.38%, 59 individuals were uninfected for both bacterial species). In the West Region, *Aedes* mosquitoes were either co-infected (75.19%, n=97) or singly infected with *Wolbachia* (24.81%, n=32). For *Culex* genera mosquitoes, the vast majority (97.96%, n=48) were infected with *Wolbachia* only. Results of Fisher’s exact *post hoc* tests (P<0.05 significance threshold) indicated no significant associations were present in our data. As *Aedes* collections in the West Region were heavily dominated by *Ae. africanus* - a vector of YFV in forest and rainforest regions in Sub-Saharan Africa ^46^, we compared *Wolbachia* and *Asaia* prevalence rates for pooled mono-specific RNA samples (n=97) to look for any potential co-infections within this species. *Wolbachia* was detected in 100.00% of pools (97/97) and a high *Asaia* prevalence rate of 96.91% (94/97) pools was also seen, demonstrating a high likelihood of co-infections occurring in this species. However, as mono-specific pools consisting of an average of five female mosquitoes were used for analysis no statistical association analysis can be carried out. 

**Table 1.** Prevalence rates of *Wolbachia* ( *16S rRNA* gene), *Asaia* and coinfection from mosquito genera collected from the West and Central regions of Cameroon. Mosquitoes from the Central region were individually extracted and analysed. Mosquitoes from the West region were extracted from monospecific pools (same species from same collection location) and prevalence analysis represents pooled samples.

Region	Genera	*Wolbachia* (%)	*Asaia* (%)	Co-infected (%)	Uninfected (%)	Totals	Fisher's P-value
West	*Aedes*	32 (24.81)	0 (0.00)	97 (75.19)	0 (0.00)	129	>0.99
West	*Culex*	48 (97.96)	0 (0.00)	0 (0.00)	1 (2.04)	49	>0.99
Central	*Aedes*	115 (97.46)	0 (0.00)	1 (0.85)	2 (1.69)	118	>0.99
Central	*Culex*	1 (0.83)	16 (13.45)	0 (0.00)	103 (85.83)	120	>0.99
Central	*Mansonia*	0 (0.00)	39 (40.63)	0 (0.00)	57 (59.38)	96	>0.99

### Confirmation of *Wolbachia* prevalence rates through amplification of the *wsp* gene

 Our preliminary assessment of *Wolbachia* prevalence rates was generated from *16S rRNA* gene amplification. However, using the *16S rRNA* gene alone is insufficient because it can also be possible that prokaryotic *16S rRNA* genes can be amplified from dead bacterial cells ^47^ and *Wolbachia 16S rRNA* sequences has previously been detected in water containers that contained larvae of mosquitoes ^48^. We undertook further *wsp* gene amplification on a wide variety of samples collected from the West Region in which morphological identification was done to species level and individuals, of the same species were pooled into groups of up to five individuals prior to RNA extraction (monospecific pools). Screening with the *wsp* gene revealed variable estimates of *Wolbachia* prevalence rates ( Table 2). Of particular interest was the high prevalence in the most abundant species *Ae. africanus* collected in both locations in West Cameroon. A total of 341 *Ae. africanus* females from Fonakeukeu were grouped into 72 pools and 65/72 pools (90.3%) were *Wolbachia*-positive based on strong amplification of the *wsp* gene. Similarly, 34/46 pools (73.9%) of the pools, representing a total of 228 *Ae. africanus* females from Toutsang amplified the *wsp* gene. Variable prevalence rates were also seen in other morphologically identified species, including *wsp* amplification in species within the *Culex*, *Aedes*, *Mansonia*, *Uranotaenia* and *Eretmapodites* genera.

**Table 2.** *Wolbachia* infection prevalence using the *wsp* gene. Based on morphological identification to genera/species and *wsp* gene amplificaiton in mosquitoes collected from the West Region of Cameroon.

Collection site	*Genera*	Species(number)	*wsp*+/totalpools (%)
Fonakeukeu	*Aedes*	*africanus (341)*	65/72 (90.3%)
Fonakeukeu	*Aedes*	*argenteopunctatus (1)*	1/1 (100%)
Fonakeukeu	*Aedes*	*tarsalis (3)*	1/3 (33.3%)
Fonakeukeu	*Culex*	unknown (5)	0/5 (0.0%)
Fonakeukeu	*Culex*	*moucheti (25)*	5/8 (62.5%)
Fonakeukeu	*Culex*	*ornathotoracis (4)*	0/4 (0.0%)
Fonakeukeu	*Culex*	*tigripes (2)*	1/1 (100%)
Fonakeukeu	*Culex*	*univitattus (4)*	1/3 (33.3%)
Fonakeukeu	*Culex*	*wigglesworthi (7)*	1/3 (33.3%)
Fonakeukeu	*Mansonia*	*maculipennis (1)*	0/1 (0.0%)
Fonakeukeu	*Mansonia*	*annetii (1)*	0/1 (0.0%)
Fonakeukeu	*Eretmapodites*	*chrysogaster var (2)*	1/3 (33.3%)
Fonakeukeu	*Uranotaenia*	*billineata connali (6)*	2/4 (50%)
Toutsang	*Aedes*	*africanus (228)*	34/46 (73.9%)
Toutsang	*Aedes*	*tarsalis (2)*	0/1 (0.0%)
Toutsang	*Aedes*	unknown (1)	1/1 (100%)
Toutsang	*Aedes*	*circumluteolus (1)*	1/1 (100%)
Toutsang	*Aedes*	*fraseri (1)*	1/1 (100%)
Toutsang	*Aedes*	*gibbinsi (3)*	1/1 (100%)
Toutsang	*Culex*	*unknown (14)*	2/5 (40.0%)
Toutsang	*Culex*	*moucheti (28)*	2/5 (40.0%)
Toutsang	*Culex*	*tigripes (7)*	0/4 (0.0%)
Toutsang	*Culex*	*univitattus (7)*	1/2 (50.0%)
Toutsang	*Culex*	*duttoni (4)*	1/3 (33.3%)
Toutsang	*Mansonia*	*maculipennis (2)*	0/1 pool (0.0%)
Toutsang	*Mansonia*	*annetii (1)*	0/1 pool (0.0%)
Toutsang	*Eretmapodites*	*chrysogaster var (4)*	2/3 pools (66.7%)

### Molecular species identification of selected *Wolbachia*-infected mosquito samples

**Figure 3.**
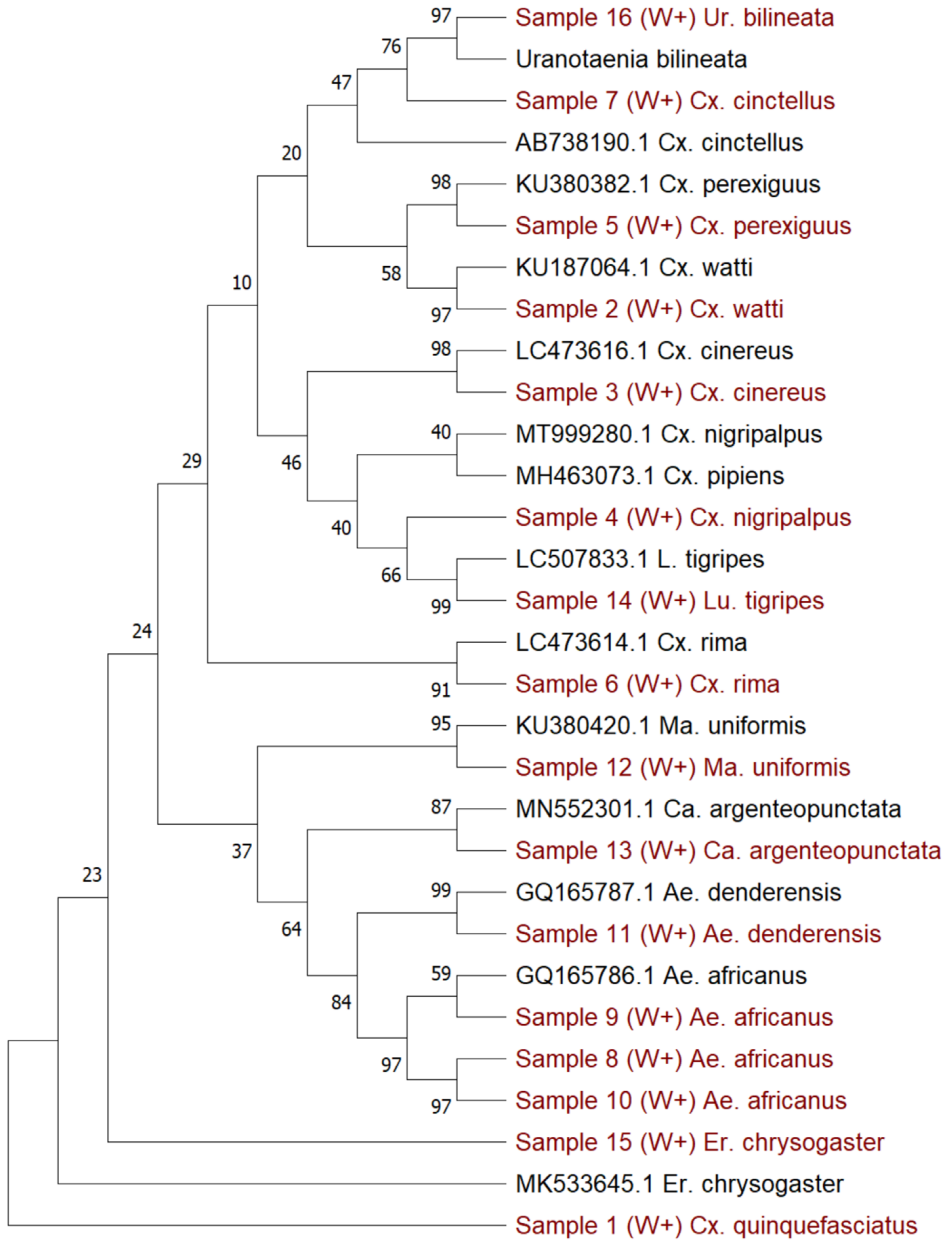
*CO1* gene phylogenetic analysis of mosquito species collected from Cameroon. Maximum Likelihood phylogenetic analysis using the General Time Reversible model. A discrete Gamma distribution was used to model evolutionary rate differences among sites (5 categories (+ *G*, parameter = 0.6443)). The tree comprises 30 nucleotide sequences with 725 positions in the dataset. Drawn to a 0.05 scale.

After using the *16S rRNA* and *wsp* genes to provide a preliminary indication of infection status, *COI* gene barcoding ^41^ was done to molecularly identify the species of a sub-sample of mosquitoes, given the difficulties associated with morphological identification of less well-studied species ( Table 3– Table 4, Figure 3). Our results showed that within these 13 sub-selected *Wolbachia*-infected samples, there were seven *Culex* species, three *Aedes* species and one species each of five additional genera, confirmed to species level using Sanger sequencing of the *COI* gene: *Cx. quinquefasciatus*, *Cx. watti*, *Cx. cinereus, Cx. nigripalpus, Cx. perexiguus, Cx. rima*, *Cx. cinctellus, Ae. africanus, Ae. denderensis, Ma. uniformis, Ca. argenteopunctata, Lu. tigripes, Er. chrysogaster* and *Ur. bilineata* ( Table 2). To differentiate between sibling species within the *Cx. pipiens* complex, we amplified the *ACE1* gene and gel electrophoresis indicated a band size of 274 base pairs, which is diagnostic for *Cx. quinquefasciatus*. To avoid potentially mis-labelling species without sufficient sequence similarity, samples with species identity below 94% were designated ‘ *cf*’ as this would be more indicative of a species that is closely related. However, the lack of sequences available for many of these species could result in genetic variation within the same species accounting for lower-than-expected sequence similarities.

**Table 3.** *CO1* gene sanger sequencing for molecular confirmation of mosquito species. The NCBI BLAST percentage (%) identity and coverage are shown alongside the closest NBCI accession number (no.) and associated species. For identity 94% and under ‘ *cf*’ has been added given the uncertainty of species identification.

Sample	Collection site	identity (%)	coverage (%)	NCBI accession number	Species
S1	Yaoundé	99	99	MK300247.1	*Cx. quinquefasciatus*
S2	Olama village	98	91	KU187063.1	*Cx. watti*
S3	Toutsang	97	100	LC473616.1	*Cx. cinereus*
S4	Toutsang	94	100	MT999280.1	*Cx. cf nigripalpus*
S5	Fonakeukeu	98	99	KU380382.1	*Cx. perexiguus*
S6	Fonakeukeu	94	99	LC473614.1	*Cx. cf rima*
S7	Olama	95	89	AB738190.1	*Cx. cinctellus*
S8	Toutsang	95	100	GQ165786.1	*Ae. africanus*
S9	Toutsang	94	100	GQ165786.1	*Ae. cf. africanus*
S10	Toutsang	94	100	GQ165786.1	*Ae. cf. africanus*
S11	Fonakeukeu	99	97	GQ165787.1	*Ae. denderensis*
S12	Olama village	99	93	KU380420.1	*Ma. uniformis*
S13	Fonakeukeu	94	100	MN552301.1	*Ca. cf. argenteopunctata*
S14	Toutsang	100	99	LC507833.1	*Lu. tigripes*
S15	Toutsang	90	99	MK533645.1	*Er. cf. chrysogaster*
S16	Toutsang	99	99	LC473729.1	*Ur. bilineata*

**Table 4.** *CO1* and *Wolbachia 16S rRNA* GenBank accession numbers. Location, species and sample code are shown alongside Genbank accession numbers. Sample sequences without accession numbers were of insufficient quality to obtain GenBank accession numbers.

Location	Sample ID	Species	*CO1* accession number	*16S rRNA* accession number
Yaoundé	S1	*Cx. quinquefasciatus*		OP745953
Olama village	S2	*Cx. watti*	OP744462	
Toutsang	S3	*Cx. cinereus*	OP744463	
Toutsang	S4	*Cx. cf nigripalpus*	OP744465	OP746031
Fonakeukeu	S5	*Cx. perexiguus*	OP744466	OP746061
Fonakeukeu	S6	*Cx. cf rima*	OP744493	OP746056
Olama	S7	*Cx. cinctellus*		OP746069
Toutsang	S8	*Ae. africanus*	OP744519	OP746071
Toutsang	S9	*Ae. cf. africanus*	OP744523	OP747286
Toutsang	S10	*Ae. cf. africanus*		OP750996
Fonakeukeu	S11	*Ae. denderensis*	OP744531	OP747294
Olama village	S12	*Ma. uniformis*	OP744580	OP747304
Fonakeukeu	S13	*Ca. cf. argenteopunctata*	OP744988	OP747416
Toutsang	S14	*Lu. tigripes*	OP745009	OP747419
Toutsang	S15	*Er. cf. chrysogaster*	OP745018	OP747455
Toutsang	S16	*Ur. bilineata*	OP745056	OP747456

### *Wolbachia* genetic diversity and MLST gene allelic profiling

**Figure 4.**
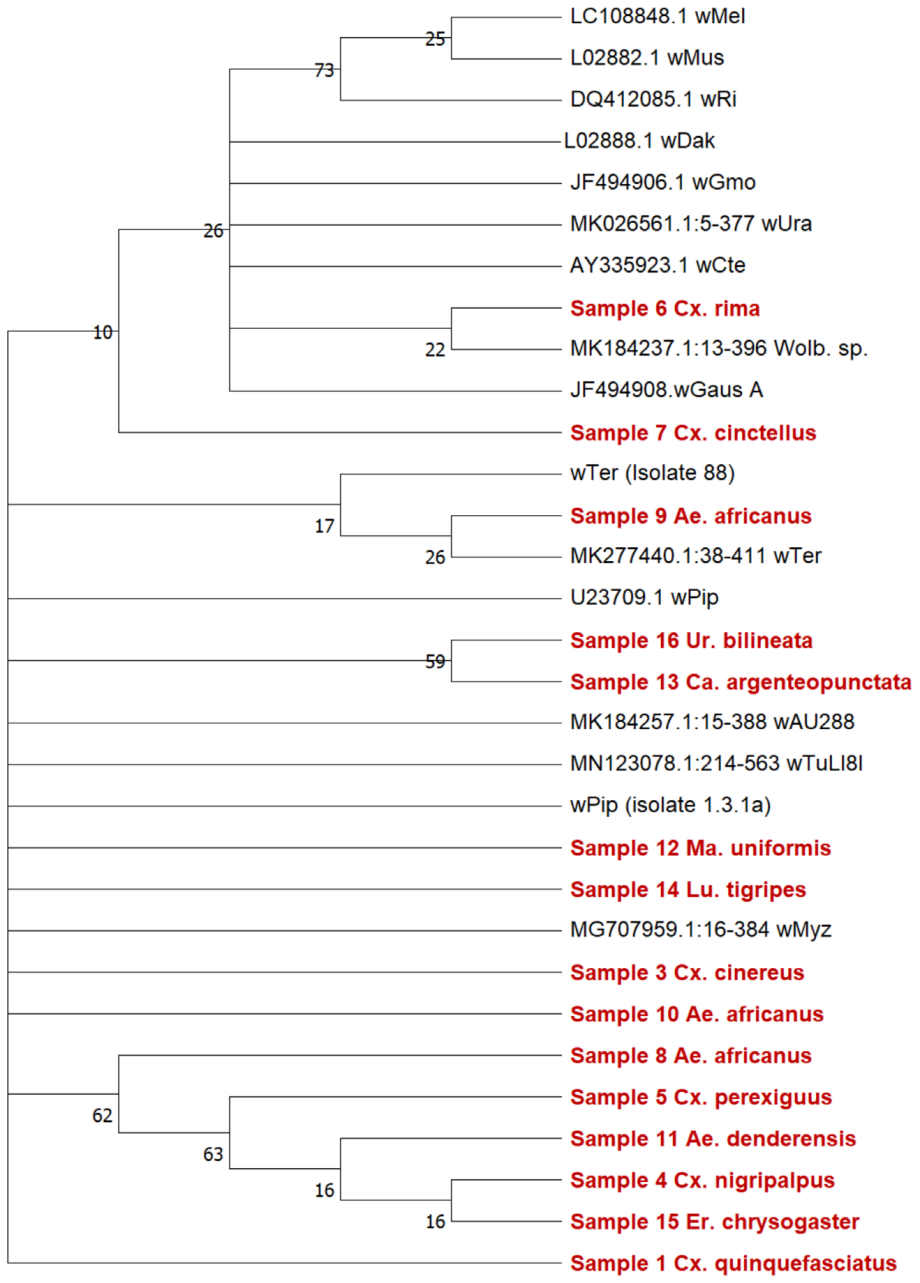
*16S rRNA* gene phylogenetic analysis of *Wolbachia* strains. The tree was produced using the Maximum Likelihood method and Jukes-Cantor model. The tree contains 31 nucleotide sequences and 1610 positions in the dataset. *Wolbachia* strains detected in this study are highlighted in red, sequences from additional strains obtained from Genbank with accession numbers are shown in black. Drawn to a 0.05 scale.

We used *16S rRNA* phylogeny to put the strains detected in this study into context with existing strains ( Figure 4). Our results showed eight strains are clustering closely together. In addition, there is sequence diversity among strains found infecting *Ae. africanus* (samples 8–10). An in-depth analysis was undertaken through MLST gene allelic profiling ( Table 5) from representatives of each mosquito species from *wsp*-positive individuals (Central Region) or monospecific pools (West Region) after species identification was confirmed. Complete MLST sequences are present for *Cx. quinquefasciatus, Cx. watti, Ae. africanus* (Sample 8) and *Ca. argenteopunctata.* The remaining samples had sequences of sufficient quality from 2–4 genes. For example, we were only able to obtain MLST gene sequences for two genes for *Ma. uniformis (gatB* and *coxA*). All sequences of sufficient quality were submitted to Genbank to obtain accession numbers Table 6.

**Table 5.** MLST gene allelic profiling. Assigned allele numbers matching those present in the *Wolbachia* MLST database ( https://pubmlst.org/organisms/wolbachia-spp/) indicating nucleotide differences in brackets.

Sample ID	Mosquito species	*gatB*	*coxA*	*hcpA*	*ftsZ*	*fbpA*
S1	*Cx. quinquefasciatus*	4	3	3 (1)	241	4
S2	*Cx. watti*	9	14 (2)	12	7	203 (1)
S3	*Cx. cinereus*	71	11	303	-	90
S4	*Cx. cf nigripalpus*	1	1	1	3	1
S5	*Cx. perexiguus*	-	11	74	-	6
S6	*Cx. cf rima*	22	2	74	258	-
S7	*Cx. cinctellus*	71	11	303	-	90
S8	*Ae. africanus*	9	8	74	106	6
S9	*Ae. cf. africanus*	9	8	74	117	203
S10	*Ae. cf. africanus*	9	8	12	117	203
S11	*Ae. denderensis*	9	11	74	106	6
S12	*Ma. uniformis*	9	14	-	-	43 (12)
S13	*Ca. cf. argenteopunctata*	9	11	303	7	446
S14	*Lu. tigripes*	71	-	74	117	6
S15	*Er. cf. chrysogaster*	-	275	-	106	6
S16	*Ur. bilineata*	9	11	74	-	90

**Table 6.** GenBank accession numbers for MLST gene sequences. Sample sequences without accession numbers were of insufficient quality to obtain GenBank accession numbers.

Sample ID	Mosquito species	*gatB*	*coxA*	*hcpA*	*ftsZ*	*fbpA*
S1	*Cx. quinquefasciatus*	OQ236162	OQ236174	OQ236185	OQ236197	OQ236208
S2	*Cx. watti*	OQ236163	OQ236175	OQ236186	OQ236198	OQ236209
S3	*Cx. cinereus*	OQ236164	OQ236176	OQ236187		OQ236210
S4	*Cx. cf nigripalpus*	OQ236165		OQ236188	OQ236199	
S5	*Cx. perexiguus*		OQ236177	OQ236189		
S6	*Cx. cf rima*	OQ236166	OQ236178	OQ236190	OQ236200	
S8	*Cx. cinctellus*	OQ236167	OQ236179	OQ236191	OQ236201	
S9	*Ae. africanus*	OQ236168		OQ236192	OQ236202	OQ236211
S10	*Ae. cf. africanus*	OQ236169	OQ236180	OQ236193	OQ236203	OQ236212
S11	*Ae. cf. africanus*				OQ236204	OQ236213
S12	*Ae. denderensis*	OQ236170	OQ236181			
S13	*Ma. uniformis*	OQ236171	OQ236182	OQ236194	OQ236205	OQ236214
S14	*Ca. cf. argenteopunctata*	OQ236172		OQ236195	OQ236206	
S15	*Lu. tigripes*		OQ236183		OQ236207	OQ236215
S16	*Er. cf. chrysogaster*	OQ236173	OQ236184	OQ236196		OQ236216

As expected, the MLST allelic profile for *Cx. quinquefasciatus* mostly matched with strain type (ST) 9 for the *w*Pip strain which infects *Cx. pipiens* and *Cx. quinquefasciatus,* although our sample had a match to *ftsZ* allele number 241, whereas existing profiles for ST 9 had an *ftsZ* allele number of 22. However, this represents only four nucleotide differences across the 435 base pairs for *ftsZ* alleles 241 and 22 resulting in 99.1% sequence identity. In *Cx. watti*, the *Wolbachia* strain allelic profile is most similar to Supergroup B *Wolbachia* strains found in the *Coenonympha hero* (the scarce heath butterfly) *–* (ST 296), the moth *Amblyptilia punctidactyla*, *Coenonympha pamphilus* (the small heath butterfly), the Fischer’s butterfly *Tongeia fischeri* (ST 300) and the thrip *Pezothrips kellyanus* (ST 430). However, only *gatB* (allele 9) and *ftsZ* (allele 7) show exact matches to these three strain types with *hcpA* and *fbpA* alleles being variable both for the novel strain in *Cx. watti* and STs 296, 300 and 430. The allelic profile of the *Wolbachia* strain detected in *Cx. cinerus* was most similar to 11 different strain types (3,108,151,213,366,382,387,461,462,472,492) but appears a novel strain as none of these typed strains contain the combination of the four alleles identified in *Cx. cinerus* (we were unable to sequence the *ftsZ* gene). For example, ST 108 has the same gatB allele (71) but the remaining three genes for comparison ( *coxA*, *hcpA* and *fbpA*) have variable allele numbers which are not the same as those sequenced from the *Wolbachia* strain in *Cx. cinerus.*

The *Wolbachia* strain detected in *Cx. cf. nigripalpus* produced an exact match to strain type 13 which is found in numerous insect species, including *Drosophila recens* and *Leucophenga maculosa* fruit flies *and Rhagoletis cerasi (*cherry fruit fly *)*. The widespread occurrence of this *Wolbachia* strain type across multiple insect genera requires further investigation given 19 isolates are present in the database. We were only able to sequence three genes for the *Wolbachia* strain detected in *Cx. perexiguus* ( *coxA*, *fbpA* and *hcpA*) resulting in three nearest strain types (108, 187,467) of which none had allele number 11 for *coxA*. However, a complete MLST profile would help confirm what appears to be a novel strain in *Cx. perexiguus* given its unique combination of three sequenced MLST genes. Likewise, the *Wolbachia* strain detected in *Cx. rima* would appear novel given only two of four allelic loci ( *gatB* and *coxA*) matched the closest ST (52) previously reported in *Anoplolepis gracillipes* (the yellow crazy ant). 

Given that we collected large numbers of *Ae. africanus* from the West Region of Cameroon, we included three monospecific pools (A-C) for MLST allelic profiling ( Table 5– Table 6). Our results provide evidence for multiple *Wolbachia* strains through analysis indicating allelic matches for four of five MLST genes (and divergence seen in the *16S rRNA* phylogeny between samples 8–10). Our Sanger sequencing indicated no evidence for the presence of multiple strains within the same pool, but further analysis is required to further determine if superinfections can be present within individual *Ae. africanus.* A novel strain was detected in *Ae. denderensis* as only three loci *(gatB, hcpA* and *fbpA)* matched ST 467 of a *Wolbachia* strain found in *Cabera pusaria* (Common white wave moth). A complete MLST profile was generated for the *Wolbachia* strain in *Ca. argenteopunctata* which appears novel given only two loci in combination match existing strains. For *Lutzia tigripes*, we could only produce sequences for three MLST genes, but this strain also appears novel with only three of four loci matching ST 108 – a strain found in the butterfly *Brangas felderi*. Likewise, novel strains appear to be present in both *Eretmapodites chrysogaster* and *Uranotaenia bilineata* as their partial allelic profiles did not match any other strain types in the database. 

## Discussion

In Cameroon, we previously showed that the richness of mosquito species was dependent on both habitat type and seasonality ^35^. Therefore, in this current study we analysed mosquitoes from diverse environmental settings to capture as much potential diversity in both mosquito species and corresponding resident *Wolbachia* strains. We identified what appears to be either novel strains or variants of existing characterised *Wolbachia* strains in 13 different mosquito species. A natural *Wolbachia* strain in *Cx. quinquefasciatus* mostly matching ST 9 is to be expected given the *w*Pip strain is widespread in species of the *Cx. pipiens* complex ^49– 51^. Our allelic profiling indicated evidence of some genetic diversity in the *ftsZ* allele although this was only four of 435 nucleotides (99.1% sequence similarity). Interestingly, there was no evidence of *Wolbachia* in *Cx. pipiens* collected from Madagascar ^6^ despite the prevailing assumption that the *w*Pip strain widely infects both *Cx. pipiens* and *Cx. quinquefasciatus* populations. Further studies across sub-Saharan Africa are needed to determine variability in both the prevalence rates and genetic strain diversity of the *w*Pip strain in members of the *Cx. pipiens* complex, given their important role as vectors of multiple human pathogens such as West Nile virus and filarial nematodes such as *Wuchereria bancrofti*. 

Another vector of human pathogens analysed in our study was *Ae. africanus* – a major vector of YFV. Although *Ae. africanus* is considered a sylvatic vector in rural areas, recent studies have suggested it has the capacity to colonise peri-domestic and domestic habitats ^46^. Our MLST analysis suggests there are multiple *Wolbachia* strain variants present in *Ae. africanus* with variation in gene sequences in three of the five MLST genes ( Table 5). It could also be possible that resident *Wolbachia* strain superinfections occur in *Ae. africanus* as have been seen in *Ae. albopictus* ^52^. *Mansonia uniformis* has been shown to transmit numerous arboviruses, such as Murray Valley encephalitis and Ross River virus, and has been shown to be a vector of Bancroftian lymphatic filariasis in Ghana ^53^. Interestingly, although *Wolbachia* has been previously identified in this species, no complete allelic profile is present. Our results match *gatB* and *coxA* allelic loci from *Ma. uniformis* collected in Kenya ^8^ but not *fbpA*, suggesting the possibility of a different strain variant present in populations from Cameroon. The remaining novel *Wolbachia* strains that we have identified were in mosquito species that are considered either minor vectors of human disease or implicated in transmission of WNV, such as *Cx. perexiguus*, *Cx. watti*, and *Cx. rima*. 

Although we undertook molecular barcoding by sequencing the mosquito *CO1* gene to try and provide as much confidence in species identification, caution must be taken with any results as this is dependent on the availability of sequences for comparison. For example, we identified *Wolbachia* strains in multiple individuals in which sequence identity was only 94%, suggesting these may be closely related species to the closest match sequence available on GenBank. Sample 7 was identified as *Cx. cinctellus* but with only 95% identity and 89% coverage, indicating this could also be another closely-related species. The inability to accurately identify mosquito species using molecular barcoding is common for species in which few sequences have previously been made available in databases such as GenBank. However, providing the *CO1* sequences will inform future studies looking at closely related species. 

Our results comparing *Wolbachia* and *Asaia* prevalence rates across major Culicine genera indicated a significant association only in *Mansonia* mosquitoes. High levels of co-infections (particularly within *Ae. africanus*, which dominated our collections from the West Region) add to growing evidence that *Wolbachia* and *Asaia* can co-exist in wild mosquitoes ^3^ despite studies clearly demonstrating an antagonistic association in lab colonies ^25, 26^. As *Asaia* can be acquired from the environment throughout the mosquito life cycle, the collection location becomes a significant factor that complicates this tripartite association. Larger cohort collections of mosquitoes from diverse environmental settings will provide further insight into how these two widespread species co-exist in different mosquito species.

*Wolbachia* MLST gene allelic profiling was undertaken to provide more assurances on detection of genuine endosymbiotic strains found in wild mosquito populations. However, defining whether a novel strain exists based only on PCR amplification of genes is problematic given the numerous examples of environmental contamination or host genome integration ^47, 48^. Caution must be taken when extrapolating PCR amplification to indicate the presence of a living endosymbiotic bacterium – particularly so when only a few gene targets such as *16S rRNA* are amplified and sequenced. It has been shown that *16S rRNA* prokaryotic DNA can be amplified from dead cells ^47^ and *Wolbachia 16S rRNA* can be detected just from water that previously contained mosquito larvae ^48^. If possible, extraction of mosquito RNA (as carried out in this study) to confirm expression of *Wolbachia* genes provides further evidence ^3^. Once novel strains are detected using MLST profiling, further studies are needed to confirm a genuine stable endosymbiotic association is present with the mosquito host species. This is important when low prevalence rates are detected given this may otherwise suggest that the *Wolbachia* strain is not inducing CI. Furthermore, there are several additional experiments that can be undertaken to provide further confirmation of resident *Wolbachia* strains in mosquitoes. Methods that can visualise *Wolbachia* bacteria in mosquito tissues using microscopy, such as fluorescent *in situ* hybridization, and *Wolbachia* genome sequencing, to compare genome depth and coverage of novel strains to those of other known infections, should be carried out to fully characterise novel *Wolbachia* strains. 

## Conclusions

Novel *Wolbachia* strains in Culicine mosquitoes collected from ecologically diverse settings in Cameroon add to the diversity of this highly prevalent endosymbiont in insect populations. Resident *Wolbachia* strains should be further characterised to determine the tissue tropism and density of newly discovered strains. Our study also suggests that co-infection with environmentally acquired *Asaia* bacteria is widespread in wild mosquito populations and the antagonistic relationship observed in lab colonies may not be present in wild Culicine populations. Novel *Wolbachia* strains could be considered as candidate strains for biocontrol strategies given their ability to reside naturally within existing mosquito populations and co-exist with environmentally acquired *Asaia* bacteria.

## Data Availability

GenBanK: Wolbachia endosymbiont of Culex pipiens isolate 1 16S ribosomal RNA gene, partial sequence. Accession number OP745953; https://identifiers.org/insdc:OP745953 ^54^ GenBanK: Culex watti isolate 1 cytochrome c oxidase subunit I (COX1) gene, partial cds; mitochondrial. Accession number OP744462; https://identifiers.org/insdc:OP744462 ^55^ Additional *CO1, Wolbachia 16S* gene GenBank accession numbers are listed in Table 4; GenBank: Wolbachia pipientis isolate S1 glutamyl-tRNA(Gln)amidotransferase subunit B (gatB) mRNA, partial cds. Accession number OQ236162; https://identifiers.org/insdc:OQ236162 ^56^ GenBank: Wolbachia pipientis isolate S1 cytochrome c oxidase subunit I (coxA) mRNA, partial cds. Accession number OQ236174; https://identifiers.org/insdc: OQ236174 ^57^ Additional *Wolbachia* multi-locus sequence typing genes GenBank accession numbers are listed in Table 6. Open Science Framework: Diverse Novel Wolbachia strains in Culicine mosquitoes from ecologically diverse regions of Cameroon, https://doi.org/10.17605/OSF.IO/V75DU This project contains the raw PCR screening data. Data are available under the terms of the Creative Commons Zero “No rights reserved” data waiver (CC0 1.0 Public domain dedication).
