# Supplementary material for: Diverse novel Wolbachia bacteria strains and genera-specific co-infections with Asaia bacteria in Culicine mosquitoes from ecologically diverse regions of Cameroon
Source: Wellcome Open Res. 2023 Sep 26;8:267. Originally published 2023 Jun 21. [Version 2] doi: 10.12688/wellcomeopenres.18580.2 (PMC10548110; doi:10.12688/wellcomeopenres.18580.2)
Supplement: Peer review history

# Reviewer report: Reviewer response for version 2

**DOI:** 10.21956/wellcomeopenres.22165.r67662

**Published:** 2023-10-10

**Authors:** Eric P. Caragata

**Affiliations:** 1 Florida Medical Entomology Laboratory, Department of Entomology and Nematology, Institute of Food and Agricultural Sciences, University of Florida, Gainesville, Florida, USA

All of my previous comments have been addressed to my satisfaction. The only other note I want to give is that it could be beneficial to explicitly delineate methodological differences in the handling of Western and Central region mosquitoes in the methods section.

Is the work clearly and accurately presented and does it cite the current literature?

Yes

If applicable, is the statistical analysis and its interpretation appropriate?

Partly

Are all the source data underlying the results available to ensure full reproducibility?

Yes

Is the study design appropriate and is the work technically sound?

Yes

Are the conclusions drawn adequately supported by the results?

Yes

Are sufficient details of methods and analysis provided to allow replication by others?

Partly

Reviewer Expertise:

Mosquito microbiology, molecular biology, immunity, and metabolism. Wolbachia. Mosquito-microbe-pathogen interactions.

I confirm that I have read this submission and believe that I have an appropriate level of expertise to confirm that it is of an acceptable scientific standard.

# Reviewer report: Reviewer response for version 2

**DOI:** 10.21956/wellcomeopenres.22165.r67661

**Published:** 2023-10-06

**Authors:** Guido Favia

**Affiliations:** 1 School of Bioscience and Veterinary Medicine, University of Camerino, Camerino, Italy

I have read the revised version of the manuscript and in my opinion, the authors improved the its quality well addressing the concerns raised at the first evaluation. Consequently the manuscript is worthy for indexing.

Is the work clearly and accurately presented and does it cite the current literature?

Partly

If applicable, is the statistical analysis and its interpretation appropriate?

I cannot comment. A qualified statistician is required.

Are all the source data underlying the results available to ensure full reproducibility?

Yes

Is the study design appropriate and is the work technically sound?

Partly

Are the conclusions drawn adequately supported by the results?

Partly

Are sufficient details of methods and analysis provided to allow replication by others?

Yes

Reviewer Expertise:

NA

I confirm that I have read this submission and believe that I have an appropriate level of expertise to confirm that it is of an acceptable scientific standard.

# Reviewer report: Reviewer response for version 2

**DOI:** 10.21956/wellcomeopenres.22165.r67660

**Published:** 2023-10-03

**Authors:** Perran Ross

**Affiliations:** 1 The University of Melbourne, Melbourne, Victoria, Australia

The authors have substantially revised the manuscript and all issues raised have been sufficiently addressed. I have no further suggestions.

Is the work clearly and accurately presented and does it cite the current literature?

Partly

If applicable, is the statistical analysis and its interpretation appropriate?

Partly

Are all the source data underlying the results available to ensure full reproducibility?

Partly

Is the study design appropriate and is the work technically sound?

Partly

Are the conclusions drawn adequately supported by the results?

Partly

Are sufficient details of methods and analysis provided to allow replication by others?

Partly

Reviewer Expertise:

Wolbachia infections in Aedes mosquitoes and their use in control programs

I confirm that I have read this submission and believe that I have an appropriate level of expertise to confirm that it is of an acceptable scientific standard.

# Reviewer report: Reviewer response for version 1

**DOI:** 10.21956/wellcomeopenres.20603.r61513

**Published:** 2023-08-01

**Authors:** Perran Ross

**Affiliations:** 1 The University of Melbourne, Melbourne, Victoria, Australia

**General comments**

 Wolbachia endosymbionts are prevalent in insect populations and can enact dramatic effects on their hosts. Understanding the prevalence and diversity of Wolbachia in natural mosquito populations has important implications for the control of mosquito-borne disease because Wolbachia infections can influence mosquito vectorial capacity and modulate the success of vector control programs. Other microbes such as Asaia can also reside in mosquito tissues where they can influence host phenotypes and interact with Wolbachia. Here, the authors performed field surveys of Culicine mosquitoes in two regions of Cameroon and screen either individuals or pools of mosquitoes for Wolbachia and Asaia using PCR detection and sequencing of 16S rRNA, wsp and MLST markers. They detect Wolbachia and Asaia at variable frequencies which depend on the mosquito genus and region. While these results provide important data for vector control programs and may lead to candidate strains for Wolbachia release programs, there are a few key limitations with their study design and their conclusions are overstated.

 The authors claim to detect co-infections of Wolbachia and Asaia in field-collected mosquitoes, contradicting evidence from laboratory studies that the two microbes have an antagonistic relationship with each other. However, these two observations are not necessarily in conflict. While there may be mutual exclusion of Asaia and Wolbachia in the reproductive tissues of some mosquitoes, this does not preclude the two microbes from residing in the same mosquito but within different tissues. There are several cases where Wolbachia and Asaia have been detected in the same mosquito (e.g. Schrieke et al. 2022 Computational and Structural Biotechnology ^1^, Hegde et al. 2018 Frontiers in Microbiology ^2^, da Silva et al. 2022 genes ^3^, Chen et al. 2020 Frontiers in Microbiology ^4^) but they may not be occupying the same tissues, especially given that Asaia can be environmentally acquired and often resides in the gut. This study also does not provide sufficient evidence for a Wolbachia-Asaia coinfection. Firstly, all samples where both Wolbachia and Asaia were detected are from pools of mosquitoes, so it is possible that these samples contain a mix of singly infected individuals. Secondly, molecular detection is not sufficient evidence of an infection as pointed out by previous studies (Chrostek and Gerth 2019 mBio ^5^, Ross et al. 2020 Ecol Evol ^6^). This is acknowledged at the end of the discussion but the rest of the paper is written as if all these detections represent true infections.

 There are also some limitations and apparent gaps/inconsistencies in their study design. Mosquitoes from the West region were pooled while mosquitoes from the Central region were tested individually, but mosquitoes were only identified to the species level in the West region. The use of pools is likely to overestimate the prevalence of Wolbachia and Asaia in a sample given that not all individuals need to test positive for the pool to be considered positive. There is also the risk that any errors with morphological species identification could lead to an incorrect assignment of Wolbachia or Asaia status of a pool if one species is positive and the other is negative. The authors also use a mix of 16S rRNA and wsp detection for Wolbachia, but the data for 16S are combined across species within a genus while the wsp data are only presented for individual species. Furthermore, the results for 16S include only Aedes, Mansonia and Culex while there are other genera included for the wsp marker. The justification for this is unclear, and it would be useful to know how concordant the two markers are with each other, especially when the authors note that the use of the 16S rRNA gene has limitations for Wolbachia detection. The authors also mention in the methods that they also used the FtsZ marker for initial Wolbachia detection but these results are not presented.

 The authors then select a sample of mosquito species testing positive for Wolbachia for further analysis of the Wolbachia strains with 16S rRNA and MLST markers. The authors place an emphasis on the fact that the Wolbachia strains that they detect are novel and diverse but these terms are not well defined. Has Wolbachia been detected in any of these species besides Cx. quinquefasciatus before, and how different do Wolbachia sequences have to be before they are considered novel and diverse?  The authors compared their sequences to the MLST database but this may not represent the full diversity of Wolbachia strains that have been identified given that Wolbachia sequences have been obtained through other approaches (e.g. Scholz et al. Nature Communications). There are also some issues with the use of the MLST system for strain typing and diversity as outlined by Bleidorn and Gerth 2018 (FEMS Microbiol Ecol) ^7^ and these should be acknowledged.

**Specific comments**

**Title** – The title is misleading – the authors have detected Wolbachia and Asaia sequences in a range of field-collected mosquito species, but there is insufficient evidence that these represent true infections. The terms “diverse” and “novel” are not well defined and it is unclear how many of these strains have been detected for the first time.  

**Introduction** – the authors mention that identifying Wolbachia strains can provide important comparative data but they don’t elaborate on how this information would be useful for biocontrol strategies. For instance, natural infections could interfere with releases of transinfections because they may change patterns of cytoplasmic incompatibility between the released and resident mosquitoes. The authors also note that novel strains could be considered candidates for biocontrol strategies, but what properties are you looking for in a strain for it to be considered a candidate, and what steps would need to be taken for the strain to be used (e.g. introgression or microinjection into a different mosquito line, virus blocking, cytoplasmic incompatibility and so on)?

**Introduction paragraph 1** – References 11 and 12 refer to Ae. polynesiensis, not Ae. albopictus.

**Introduction paragraph 1 **– There is an emphasis on wMel here specifically, but also consider including the wAlbB strain which was the first transinfected strain in Ae. aegypti (Xi et al. 2005, Science) and which has also been released in wild mosquito populations to inhibit virus transmission (Nazni et al. 2019 Current Biology).

**Introduction paragraph 2 **– “However, a more targeted approach amplifying *Wolbachia*-specific genes is required to confirm a resident strain is present” – This really requires additional evidence such as microscopy, removal of the infection, demonstration of maternal transmission and so on.

**Introduction paragraph 2** – provide an example of a superinfection comprising strains from the same supergroup

**Introduction paragraph 3 **– “outbreaks of arboviral diseases including DENV, YFV, CHIKV and Rift

 Valley fever virus (RVFV) are a possibility.” is a bit vague – do they occur and how frequently?

**Methods **- How many mosquitoes per pool? In the results you mention an average of 5 female mosquitoes, but how much did this vary?

**Methods **– sanger sequencing – which PCR products were considered to be worthy of sequencing and why?

**Results paragraph 1 **– When presenting West region results, do these n values represent the number of pools? Or were these actually individuals?

**Results paragraph 1** - What is the hypothesis being tested with the Fisher’s exact tests? Please elaborate on what associations are being tested and include these statistical tests in the methods section

**Results** – There appear to be some inconsistencies when presenting frequency data- sometimes samples from the West region are specifically referred to as pools while at other times there is no mention of them being individuals or pools (e.g. in the first paragraph of the results).

**Table 1 **– results for Wolbachia 16S are reported, but what about other genes? Were they concordant with the 16S results?

**Table 1 **– What is the Fisher test comparing?

**Table 1** – Although mentioned as a caveat in the text, it is misleading to put “co-infection” for the West samples when these consist of pools of mosquitoes – it is plausible that pools could consist of singly infected Wolbachia and Asaia individuals

**Table 2** – Only the last three rows indicate that pools were tested, but aren’t all samples in this table pools?

**Figure 1** – Please provide the 16S data by species to allow for a comparison with the wsp data

**Results **- *"Wolbachia 16S rRNA sequences has previously been detected in water containers that contained larvae of mosquitoes*” - Is this not also possible for the other genes?

**Discussion** – What makes a strain a candidate? Is any newly detected strain a candidate strain, or do they need to display a certain phenotype such as cytoplasmic incompatibility or virus blocking? In my opinion a candidate strain must possess some desirable traits and should be able to be maintained in the laboratory.

Is the work clearly and accurately presented and does it cite the current literature?

Partly

If applicable, is the statistical analysis and its interpretation appropriate?

Partly

Are all the source data underlying the results available to ensure full reproducibility?

Partly

Is the study design appropriate and is the work technically sound?

Partly

Are the conclusions drawn adequately supported by the results?

Partly

Are sufficient details of methods and analysis provided to allow replication by others?

Partly

Reviewer Expertise:

Wolbachia infections in Aedes mosquitoes and their use in control programs

I confirm that I have read this submission and believe that I have an appropriate level of expertise to state that I do not consider it to be of an acceptable scientific standard, for reasons outlined above.

## References

[1] : The mosquito microbiome includes habitat-specific but rare symbionts. *Comput Struct Biotechnol J* .2022;20: 10.1016/j.csbj.2021.12.019 410-420 10.1016/j.csbj.2021.12.019 35140881PMC8803474

[2] : Microbiome Interaction Networks and Community Structure From Laboratory-Reared and Field-Collected Aedes aegypti, Aedes albopictus, and Culex quinquefasciatus Mosquito Vectors. *Front Microbiol* .2018;9: 10.3389/fmicb.2018.02160 2160 10.3389/fmicb.2018.02160 30250462PMC6140713

[3] : Bacterial Community Diversity and Bacterial Interaction Network in Eight Mosquito Species. *Genes (Basel)* .2022;13(11) : 10.3390/genes13112052 10.3390/genes13112052 36360289PMC9690548

[4] : Multiple Factors Determine the Structure of Bacterial Communities Associated With Aedes albopictus Under Artificial Rearing Conditions. *Front Microbiol* .2020;11: 10.3389/fmicb.2020.00605 605 10.3389/fmicb.2020.00605 32351473PMC7176356

[5] : Is Anopheles gambiae a Natural Host of Wolbachia?. *mBio* .2019;10(3) : 10.1128/mBio.00784-19 10.1128/mBio.00784-19 31186318PMC6561020

[6] : An elusive endosymbiont: Does Wolbachia occur naturally in Aedes aegypti?. *Ecol Evol* .2020;10(3) : 10.1002/ece3.6012 1581-1591 10.1002/ece3.6012 32076535PMC7029055

[7] : A critical re-evaluation of multilocus sequence typing (MLST) efforts in Wolbachia. *FEMS Microbiol Ecol* .2018;94(1) : 10.1093/femsec/fix163 10.1093/femsec/fix163 29186405

# Response

**Published:** 2023-09-04

**Authors:** Thomas Walker

**Affiliations:** University of Warwick, UK

Dear Perran, **Thank you so much for your very comprehensive review. We have addressed your comments as follows with responses in bold: ** Wolbachia endosymbionts are prevalent in insect populations and can enact dramatic effects on their hosts. Understanding the prevalence and diversity of Wolbachia in natural mosquito populations has important implications for the control of mosquito-borne disease because Wolbachia infections can influence mosquito vectorial capacity and modulate the success of vector control programs. Other microbes such as Asaia can also reside in mosquito tissues where they can influence host phenotypes and interact with Wolbachia. Here, the authors performed field surveys of Culicine mosquitoes in two regions of Cameroon and screen either individuals or pools of mosquitoes for Wolbachia and Asaia using PCR detection and sequencing of 16S rRNA, wsp and MLST markers. They detect Wolbachia and Asaia at variable frequencies which depend on the mosquito genus and region. While these results provide important data for vector control programs and may lead to candidate strains for Wolbachia release programs, there are a few key limitations with their study design and their conclusions are overstated.

 The authors claim to detect co-infections of Wolbachia and Asaia in field-collected mosquitoes, contradicting evidence from laboratory studies that the two microbes have an antagonistic relationship with each other. However, these two observations are not necessarily in conflict. While there may be mutual exclusion of Asaia and Wolbachia in the reproductive tissues of some mosquitoes, this does not preclude the two microbes from residing in the same mosquito but within different tissues. There are several cases where Wolbachia and Asaia have been detected in the same mosquito (e.g. Schrieke et al. 2022 Computational and Structural Biotechnology ^1^ , Hegde et al. 2018 Frontiers in Microbiology ^2^ , da Silva et al. 2022 genes ^3^ , Chen et al. 2020 Frontiers in Microbiology ^4^ ) but they may not be occupying the same tissues, especially given that Asaia can be environmentally acquired and often resides in the gut. **Thank you for these points.  We have expanded paragraph 4 of our discussion to provide a more balanced view on the relationship between *Wolbachia* and *Asaia*. ** This study also does not provide sufficient evidence for a Wolbachia-Asaia coinfection. Firstly, all samples where both Wolbachia and Asaia were detected are from pools of mosquitoes, so it is possible that these samples contain a mix of singly infected individuals. **We have added a caveat to our discussion on ‘pooling’ mosquitoes (paragraph 4) but given that you have pointed out that ‘there are several cases where Wolbachia and Asaia have been detected in the same mosquito’ it would be contradictory to those studies to assume a coinfection is not possible. Unfortunately these collections were undertaken during the covid pandemic so a pooling approach was taken to minimise time in labs under severe travel restrictions.  Our data from the West region ( *Aedes* monospecific pools mosquitoes were either co-infected (75%) or singly infected with *Wolbachia* (25%) and for *Culex* 98% were infected with *Wolbachia* only) would also indicate a mix of singly infected individuals is not likely to represent most of our data. ** Secondly, molecular detection is not sufficient evidence of an infection as pointed out by previous studies (Chrostek and Gerth 2019 mBio ^5^ , Ross et al. 2020 Ecol Evol ^6^ ). This is acknowledged at the end of the discussion but the rest of the paper is written as if all these detections represent true infections. **We agree there have been many recent papers that have just presented molecular data without providing further evidence but we feel our manuscript provides sufficient caveats to justify our results as more than likely coming from genuine infections (paragraphs 4 and 5 of the discussion). We acknowledge that reliance on just one *Wolbachia* gene (eg. *16S rRNA* or wsp) would not be sufficient as has been the case for numerous recent publications. However, having multiple MLST gene sequences from *Wolbachia* housekeeping genes that match (or are very close in sequence) to existing sequencing in** **https://pubmlst.org/organisms/wolbachia-spp** **from RNA extracts (so active gene expression) would indicate it’s much more likely to represent genuine strains than the alternatives such as environmental contamination.  We support the general opinion that further experimental work is needed but methods such as whole genome sequencing and FISH are not feasible on large numbers of field collected mosquitoes (from different species) without prior knowledge of molecular evidence that strain exist (or are likely to exist based on high prevalence rates and consistent strain typing).  ** There are also some limitations and apparent gaps/inconsistencies in their study design. Mosquitoes from the West region were pooled while mosquitoes from the Central region were tested individually, but mosquitoes were only identified to the species level in the West region. **Unfortunately we were unable to morphologically identify species in the Central region due to 1) a lack of expertise and 2) missing or damaged morphological features during mosquito collections.  Morphological identification of less-well known Culicine species is particularly challenging and often results in misidentification** **^1^** **. Therefore, we firstly screened individuals for symbionts using qPCR targeting both *Wolbachia* and *Asaia* *16S rRNA* genes to identify samples of interest (and limited our analysis to the genera level). MLST sequencing was then carried out on selected individuals for *Wolbachia* strain typing (our main aim of this work) followed by molecular confirmation of mosquito species using CO1 barcoding.  ** The use of pools is likely to overestimate the prevalence of Wolbachia and Asaia in a sample given that not all individuals need to test positive for the pool to be considered positive. There is also the risk that any errors with morphological species identification could lead to an incorrect assignment of Wolbachia or Asaia status of a pool if one species is positive and the other is negative. **We again agree that pooling is not the preferred method for detection but covid restrictions (both in the field for travel and in the lab for analysis) resulted in a pooling strategy to be able to analyse samples.  We have added a caveat to our discussion on ‘pooling’ mosquitoes (paragraph 4) to address this point. ** The authors also use a mix of 16S rRNA and wsp detection for Wolbachia, but the data for 16S are combined across species within a genus while the wsp data are only presented for individual species.Furthermore, the results for 16S include only Aedes, Mansonia and Culex while there are other genera included for the wsp marker. The justification for this is unclear, and it would be useful to know how concordant the two markers are with each other, especially when the authors note that the use of the 16S rRNA gene has limitations for Wolbachia detection. The authors also mention in the methods that they also used the FtsZ marker for initial Wolbachia detection but these results are not presented. **Please see our previous response in terms of the difficulties of morphological ID from the Central region (lack of expertise and damage from collections).  We used the wsp gene on our pooled samples from the West to re-enforce what appeared to be a high prevalence rate from the monospecific *Aedes* pools.  In contrast to 16S rRNA, wsp is often not amplified from low density strains (or strains that may not be in genuine endosymbiosis).  For example, there are numerous studies that report the presence of Wolbachia in Anopheles species in which wsp failed to amplify (PMID:** **32787974). Genbank lists 835 sequences for wsp (search wsp AND "Wolbachia pipientis"[porgn:__txid955]) compared to 2643 for 16S rRNA (search 16S rRNA AND "Wolbachia pipientis"[porgn:__txid955]) suggesting wsp amplification is more indicative of a genuine symbiotic association (and hypervariable regions can be used for strain typing in addition to MLST). ** The authors then select a sample of mosquito species testing positive for Wolbachia for further analysis of the Wolbachia strains with 16S rRNA and MLST markers. The authors place an emphasis on the fact that the Wolbachia strains that they detect are novel and diverse but these terms are not well defined. **We have added a sentence to our discussion (paragraph 5) on what criteria we used based on MLST. ** Has Wolbachia been detected in any of these species besides Cx. quinquefasciatus before, and how different do Wolbachia sequences have to be before they are considered novel and diverse? **This was partly addressed in our discussion for *Mansonia* but we have now checked the most reliable source for strain typing (https://pubmlst.org/organisms/wolbachia-spp) and can confirm (based on this) that no other Culex species in our study has MLST or wsp sequences.  There are also no strain profiles present for *Aedes africanus* or Aedes *denderensis or the remaining samples (Catageiomyia argenteopunctata, Lutzia tigripes, Eretmapodites chrysogaster *and *Uranotaenia bilineata). *We have added a sentence to the end of paragraph 2 of the discussion to address this. ** The authors compared their sequences to the MLST database but this may not represent the full diversity of Wolbachia strains that have been identified given that Wolbachia sequences have been obtained through other approaches (e.g. Scholz et al. Nature Communications). There are also some issues with the use of the MLST system for strain typing and diversity as outlined by Bleidorn and Gerth 2018 (FEMS Microbiol Ecol) ^7^ and these should be acknowledged. **We have included a sentence in the discussion (paragraph 5) on the limitations of MLST despite this being widely used in the Wolbachia community for strain diversity.** 

**Specific comments**

**Title** – The title is misleading – the authors have detected Wolbachia and Asaia sequences in a range of field-collected mosquito species, but there is insufficient evidence that these represent true infections. The terms “diverse” and “novel” are not well defined and it is unclear how many of these strains have been detected for the first time. **We respectively disagree that the title should be changed to ‘sequences’ given we are presenting MLST data.  There are many published papers reporting Wolbachia strain detection with much less genetic data (sometimes based on sequences obtained from nested 16S rRNA) so this change seems inappropriate.  We have changed our title to ‘Diverse novel *Wolbachia *bacteria strains and genera-specific co-infections with *Asaia *bacteria in Culicine mosquitoes from ecologically diverse regions of Cameroon’’ to reflect genera differences for Asaia co-infections.**

**Introduction** – the authors mention that identifying Wolbachia strains can provide important comparative data but they don’t elaborate on how this information would be useful for biocontrol strategies. For instance, natural infections could interfere with releases of transinfections because they may change patterns of cytoplasmic incompatibility between the released and resident mosquitoes. The authors also note that novel strains could be considered candidates for biocontrol strategies, but what properties are you looking for in a strain for it to be considered a candidate, and what steps would need to be taken for the strain to be used (e.g. introgression or microinjection into a different mosquito line, virus blocking, cytoplasmic incompatibility and so on)? **We have added some sentences in paragraph 2 of the introduction to address this important point.**

**Introduction paragraph 1** – References 11 and 12 refer to Ae. polynesiensis, not Ae. albopictus. **Thank you for pointing this error out – we have changed this**

**Introduction paragraph 1 **– There is an emphasis on wMel here specifically, but also consider including the wAlbB strain which was the first transinfected strain in Ae. aegypti (Xi et al. 2005, Science) and which has also been released in wild mosquito populations to inhibit virus transmission (Nazni et al. 2019 Current Biology). **Thank you for this suggestion and we have added some information at the end of paragraph 1**

**Introduction paragraph 2 **– “However, a more targeted approach amplifying *Wolbachia*-specific genes is required to confirm a resident strain is present” – This really requires additional evidence such as microscopy, removal of the infection, demonstration of maternal transmission and so on. **We agree and have added a sentence to reflect this**

**Introduction paragraph 2** – provide an example of a superinfection comprising strains from the same supergroup **We have not been able to find a clear example (at least within mosquitoes) so have modified this sentence to reflect this.**

**Introduction paragraph 3 **– “outbreaks of arboviral diseases including DENV, YFV, CHIKV and Rift

 Valley fever virus (RVFV) are a possibility.” is a bit vague – do they occur and how frequently? **We have modified this and added a sentence with an example for DENV in Cameroon.**

**Methods **- How many mosquitoes per pool? In the results you mention an average of 5 female mosquitoes, but how much did this vary? **We have added this (3-5 mosquitoes/pool) in the methods**

**Methods **– sanger sequencing – which PCR products were considered to be worthy of sequencing and why? **We have modified the beginning of the this section to provide further details**

**Results paragraph 1 **– When presenting West region results, do these n values represent the number of pools? Or were these actually individuals? **We have edited this paragraph for clarity**

**Results paragraph 1** - What is the hypothesis being tested with the Fisher’s exact tests? Please elaborate on what associations are being tested and include these statistical tests in the methods section **We erroneously left out a statistics section in the methods which has now been added**

**Results** – There appear to be some inconsistencies when presenting frequency data- sometimes samples from the West region are specifically referred to as pools while at other times there is no mention of them being individuals or pools (e.g. in the first paragraph of the results). **We have added ‘pools’ were appropriate to reflect the West region data**

**Table 1 **– results for Wolbachia 16S are reported, but what about other genes? Were they concordant with the 16S results? **We used qPCR targeting the 16S rRNA gene for both *Wolbachia* and *Asaia* to generate CTs to provide a consistent comparison and did not compare to endpoint PCR results.  All raw data is available at** https://doi.org/10.17605/OSF.IO/V75DU.  

**Table 1 **– What is the Fisher test comparing? **Please see our new ‘statistical analysis’ section in the methods.  “Fisher’s exact post hoc tests in GraphPad prism version 9 (P<0.05 significance threshold) were used to determine any association between prevalence rates of *Wolbachia* and *Asaia* for each mosquito genus from the different regions (West and Central). Samples were categorised as *Wolbachia*-infected, *Asaia*-infected, co-infected or uninfected.’’** **Table 1** – Although mentioned as a caveat in the text, it is misleading to put “co-infection” for the West samples when these consist of pools of mosquitoes – it is plausible that pools could consist of singly infected Wolbachia and Asaia individuals **We agree that we need to include more caveats in our discussion (see new paragraph 4) but disrespectfully disagree for table 1 given we have the following in the legend ‘Mosquitoes from the West region were extracted from monospecific pools (same species from same collection location) and prevalence analysis represents pooled samples’ to clearly show these are pooled samples.** **Table 2** – Only the last three rows indicate that pools were tested, but aren’t all samples in this table pools? **Thank you for spotting this error – we have amended this table**

**Figure 1** – Please provide the 16S data by species to allow for a comparison with the wsp data **This is a map showing the co-ordinates of the collections and has no species data**

**Results **- *"Wolbachia 16S rRNA sequences has previously been detected in water containers that contained larvae of mosquitoes*” - Is this not also possible for the other genes? **We agree it’s theoretically possible but we feel it’s only appropriate to reference published papers.  Furthermore, 16S rRNA is often reported in papers in which no other *Wolbachia *gene amplification has been possible and given it’s extremely conserved (V3/V4 region used for microbiome analysis) is more likely to result in environmental contamination.**

**Discussion** – What makes a strain a candidate? Is any newly detected strain a candidate strain, or do they need to display a certain phenotype such as cytoplasmic incompatibility or virus blocking? In my opinion a candidate strain must possess some desirable traits and should be able to be maintained in the laboratory. **Thank you for this valid point and opinion.  Given that mosquito transinfection has been possible from closely related mosquito species (eg. wAlbB into Ae. aegypti and An. stephensi), strains that natural reside within mosquito species may provide an increased chance of successful transinfection (difficult to test given the technical difficulty of embryo microinjection). Cell line adaptation was seen as key reasons for the success of Drosophila Wolbachia strains wMel and wMelPop into *Ae. aegypti* so strains that can be firstly identified to be at high prevalence in mosquito field populations will allow further examination in terms of phenotypic effects through eg. cif gene analysis to indicate CI induction.  We agree that there are numerous candidate strains in lab colonies but temperature has recently been shown to decrease both density and virus inhibition for some Drosophila strains (eg. wMel).  Potential candidate strains that naturally exist in mosquito field populations in countries such as Cameroon with high average temperature may be less likely to see this effect.** **References **   1.         Jourdain, F., Picard, M., Sulesco, T., Haddad, N., Harrat, Z., Sawalha, S.S., Gunay, F., Kanani, K., Shaibi, T., Akhramenko, D., et al. (2018). Identification of mosquitoes (Diptera: Culicidae): an external quality assessment of medical entomology laboratories in the MediLabSecure Network. Parasit Vectors *11*, 553. 10.1186/s13071-018-3127-7.

# Reviewer report: Reviewer response for version 1

**DOI:** 10.21956/wellcomeopenres.20603.r61511

**Published:** 2023-07-13

**Authors:** Guido Favia

**Affiliations:** 1 School of Bioscience and Veterinary Medicine, University of Camerino, Camerino, Italy

The work is part of a series of works, now quite numerous, which aim to identify species of insect vectors that are infected by Wolbachia. In this sense, the research is not characterized by particular originality; nevertheless the research has some appreciable aspects as it aims to analyze several species of mosquitoes (some of which are little studied) in some areas of Cameroon, in different eco-ethological contexts. Furthermore, I appreciated that the analysis of Wolbachia distribution was carried out not exclusively through 16S amplification but also through an MLST approach resulting in phylogenetic analysis.

 That said, the manuscript has some flaws which, in my opinion, should be corrected before publication.

1. For experts in the field the decision to verify the co-presence/co-absence of Wolbachia and Asaia is quite clear but for many readers it would be useful to explain why the monitoring concerned Asaia and not other symbionts (it might help in the introduction to point out that these are two of the few symbionts for which insect and vector control approaches have already been proposed and in some cases validated in semifield and field experiments).
2. Figure 2 compares the positivity between West and East mosquitoes at the genus level. I don't understand why, given that they then move on to the analysis and definition of the species, this datum is not expressed by species; it would definitely be more informative.
3. Even the logical thread expressed in the materials and methods does not seem acceptable to me. They speak first of the monitoring of Asaia and Wolbachia and then of the definition of the host species. A stringent logic that aims to monitor the distribution of two symbionts in different mosquito species, I think, would mean proceeding first with the identification of the host-species and then with monitoring the distributions of the symbionts.
4. Another aspect that should be slightly modified concerns the conclusions: the authors state that " *Our study also suggests that co-infection with environmentally acquired Asaia bacteria is widespread in wild mosquito populations and the antagonistic relationship observed in lab colonies may not be present in wild Culicine populations.*" This is not entirely true since there are studies on some wild-species of culicine that demonstrate an almost total absence of coexistence of the two symbionts, as in the case of some invasive species of aedes. The sentence therefore should be rephrased and referred to and limited to the geographical context analyzed by the authors.

Is the work clearly and accurately presented and does it cite the current literature?

Partly

If applicable, is the statistical analysis and its interpretation appropriate?

I cannot comment. A qualified statistician is required.

Are all the source data underlying the results available to ensure full reproducibility?

Yes

Is the study design appropriate and is the work technically sound?

Partly

Are the conclusions drawn adequately supported by the results?

Partly

Are sufficient details of methods and analysis provided to allow replication by others?

Yes

Reviewer Expertise:

Molecular Entomology, Molecular Parasitology, Insect symbiosis

I confirm that I have read this submission and believe that I have an appropriate level of expertise to confirm that it is of an acceptable scientific standard, however I have significant reservations, as outlined above.

# Response

**Published:** 2023-09-04

**Authors:** Thomas Walker

**Affiliations:** University of Warwick, UK

Dear Guido, **Thank you very much for taking the time out to review our manuscript. We have addressed your comments as follows with responses in bold: ** The work is part of a series of works, now quite numerous, which aim to identify species of insect vectors that are infected by Wolbachia. In this sense, the research is not characterized by particular originality; nevertheless the research has some appreciable aspects as it aims to analyze several species of mosquitoes (some of which are little studied) in some areas of Cameroon, in different eco-ethological contexts. Furthermore, I appreciated that the analysis of Wolbachia distribution was carried out not exclusively through 16S amplification but also through an MLST approach resulting in phylogenetic analysis.

 That said, the manuscript has some flaws which, in my opinion, should be corrected before publication.

1. For experts in the field the decision to verify the co-presence/co-absence of Wolbachia and Asaia is quite clear but for many readers it would be useful to explain why the monitoring concerned Asaia and not other symbionts (it might help in the introduction to point out that these are two of the few symbionts for which insect and vector control approaches have already been proposed and in some cases validated in semifield and field experiments).

**Thank you for this suggestion. We agree and have extended our introduction (paragraph 2) to incorporate a more expansive introduction to *Asaia* and its potential role for mosquito control.**

1. Figure 2 compares the positivity between West and East mosquitoes at the genus level. I don't understand why, given that they then move on to the analysis and definition of the species, this datum is not expressed by species; it would definitely be more informative.

**Unfortunately we were unable to morphologically identify species in the Central region due to 1) a lack of expertise and 2)** **missing or damaged morphological features during mosquito collections.  Morphological identification of less-well known Culicine species is particularly challenging and often results in misidentification** **^1^** **. Therefore, we firstly** **screened individuals for symbionts using qPCR targeting both *Wolbachia* and *Asaia* *16S rRNA* genes to identify samples of interest (and limited our analysis to the genera level). MLST sequencing was then carried out on selected individuals for *Wolbachia* strain typing (our main aim of this work) followed by molecular confirmation of mosquito species using CO1 barcoding** **.  **

1. Even the logical thread expressed in the materials and methods does not seem acceptable to me. They speak first of the monitoring of Asaia and Wolbachia and then of the definition of the host species. A stringent logic that aims to monitor the distribution of two symbionts in different mosquito species, I think, would mean proceeding first with the identification of the host-species and then with monitoring the distributions of the symbionts.

**Please see our response to query 2.  We firstly identified samples of interest from the *Wolbachia/Asaia* qPCR results and then proceeded to undertake *Wolbachia* MLST strain typing and confirm species using CO1 barcoding given the difficulties of Culicine morphological identification which in a study that used an external quality assessment resulted in only 64% accuracy to the species level across 19 participant laboratories** **^1^** **.   **

1. Another aspect that should be slightly modified concerns the conclusions: the authors state that " *Our study also suggests that co-infection with environmentally acquired Asaia bacteria is widespread in wild mosquito populations and the antagonistic relationship observed in lab colonies may not be present in wild Culicine populations.*" This is not entirely true since there are studies on some wild-species of culicine that demonstrate an almost total absence of coexistence of the two symbionts, as in the case of some invasive species of aedes. The sentence therefore should be rephrased and referred to and limited to the geographical context analyzed by the authors.

**Thank you for this suggestion and we agree.  We have modified our discussion in paragraph 4 to address these very valuable points.** **References**   1.         Jourdain, F., Picard, M., Sulesco, T., Haddad, N., Harrat, Z., Sawalha, S.S., Gunay, F., Kanani, K., Shaibi, T., Akhramenko, D., et al. (2018). Identification of mosquitoes (Diptera: Culicidae): an external quality assessment of medical entomology laboratories in the MediLabSecure Network. Parasit Vectors *11*, 553. 10.1186/s13071-018-3127-7.

# Reviewer report: Reviewer response for version 1

**DOI:** 10.21956/wellcomeopenres.20603.r60867

**Published:** 2023-07-13

**Authors:** Eric P. Caragata

**Affiliations:** 1 Florida Medical Entomology Laboratory, Department of Entomology and Nematology, Institute of Food and Agricultural Sciences, University of Florida, Gainesville, Florida, USA

In their manuscript ‘Diverse novel Wolbachia bacteria strains and widespread co- infections with Asaia bacteria in Culicine mosquitoes from ecologically diverse regions of Cameroon’, the authors use RT-qPCR screening of mosquito specimens collected from two areas in Cameroon to look for evidence of novel Wolbachia infections, as well as examining patterns of *Wolbachia-Asaia* co-occurrence/co-exclusion. They observe geographic and mosquito clade-specific patterns of Wolbachia and Asaia occurrence. They then use MLST sequencing data and phylogenetics to examine the similarity of their putative, new Wolbachia strains to each other and those previously characterized. Such strains, if proven to be viable and heritable, may be useful as targets to generate novel Wolbachia transinfections, but may also provide important information on the ecology, physiology, and vector competence of several mosquito species in the region.

 The manuscript is well written and does a great job of mentioning caveats. The authors have been particularly conscious of the issue of false positive detection of Wolbachia in their choice of methodology, and in their acknowledgements that all of these putative strains must be further validated, for instance, by using imaging techniques. I have a few suggestions and clarifications for the revised version of the text:

1. The issue of false positive detection of Wolbachia in mosquitoes is intrinsic to this work and field of Wolbachia research and it could be briefly mentioned in the introduction. This will give your readers improved context for your choice of RNA-based quantification of Wolbachia.
2. Details on Wolbachia detection criteria (calling positives vs negatives) as well as the positive and negative controls used in the RT-qPCR assay have not been provided in the methods section. These details are vital to demonstrate efforts have been made to reduce false positive detection.
3. Please clarify how Fisher’s tests were used to analyze your data. These tests were not mentioned in the methods section and are applied in pairwise comparisons, which might explain why some tests in Table 1 have such high P values.
4. At the beginning of the results section it would be useful to include a brief summary of which mosquito species were collected, and where and when they were collected. The when is important given samples were collected across many years.
5. I do not see an equivalent of table 2 for the central region data. It looks as though Table 2 just deals with the western region.
6. Queries about phylogenetic trees:
**-** In Fig. 3, is there an explanation for why Cx pipiens and Cx quinquefasciatus don’t cluster together.
**- **None of the phylogenetic appear to have outgroups
**- **Fig. 4 - while it is useful to have a tree based on the Wolbachia 16s gene. It could be valuable to include an unrooted consensus tree based on the MLST data.
7. Is there a reason why Figure 2 and Table 2 collapse the prevalence of Wolbachia and Asaia to the genus level? This reduces the biological relevance of your findings as mosquitoes within a genus can fill distinct biological niches.
8. Figure 2 is not visually accessible (red/green colors).
9. The discussion describes the collection areas in Cameroon as containing a high degree of environment-driven variation. Reflecting on that, the decision to bin samples into two homogeneous regions (West vs Central) does not make sense. Are there any site-specific patterns that could be mentioned that better reflect site-to-site diversity in mosquito species/Wolbachia prevalence?
10. The discussion described Wolbachia/Asaia co-infection as being “widespread” but this does not appear to reflect the data given that Asaia was only highly prevalent in one mosquito genus.
11. If the introduction were to briefly discuss the impact of Asaia on mosquito vector competence and immunity it could add important context outlining the rationale for studying Asaia infections in mosquitoes.

Is the work clearly and accurately presented and does it cite the current literature?

Yes

If applicable, is the statistical analysis and its interpretation appropriate?

Partly

Are all the source data underlying the results available to ensure full reproducibility?

Yes

Is the study design appropriate and is the work technically sound?

Yes

Are the conclusions drawn adequately supported by the results?

Yes

Are sufficient details of methods and analysis provided to allow replication by others?

Partly

Reviewer Expertise:

Mosquito microbiology, molecular biology, immunity, and metabolism. Wolbachia. Mosquito-microbe-pathogen interactions.

I confirm that I have read this submission and believe that I have an appropriate level of expertise to confirm that it is of an acceptable scientific standard, however I have significant reservations, as outlined above.

# Response

**Published:** 2023-09-04

**Authors:** Thomas Walker

**Affiliations:** University of Warwick, UK

Dear Eric, **Thank you for your very comprehensive review. We have addressed your comments as follows with responses in bold: ** In their manuscript ‘Diverse novel Wolbachia bacteria strains and widespread co- infections with Asaia bacteria in Culicine mosquitoes from ecologically diverse regions of Cameroon’, the authors use RT-qPCR screening of mosquito specimens collected from two areas in Cameroon to look for evidence of novel Wolbachia infections, as well as examining patterns of *Wolbachia-Asaia* co-occurrence/co-exclusion. They observe geographic and mosquito clade-specific patterns of Wolbachia and Asaia occurrence. They then use MLST sequencing data and phylogenetics to examine the similarity of their putative, new Wolbachia strains to each other and those previously characterized. Such strains, if proven to be viable and heritable, may be useful as targets to generate novel Wolbachia transinfections, but may also provide important information on the ecology, physiology, and vector competence of several mosquito species in the region.

 The manuscript is well written and does a great job of mentioning caveats. The authors have been particularly conscious of the issue of false positive detection of Wolbachia in their choice of methodology, and in their acknowledgements that all of these putative strains must be further validated, for instance, by using imaging techniques. I have a few suggestions and clarifications for the revised version of the text:

1. The issue of false positive detection of Wolbachia in mosquitoes is intrinsic to this work and field of Wolbachia research and it could be briefly mentioned in the introduction. This will give your readers improved context for your choice of RNA-based quantification of Wolbachia.

**Thank you for your suggestion and we have added a sentence on this at the back end of the introduction. **

1. Details on Wolbachia detection criteria (calling positives vs negatives) as well as the positive and negative controls used in the RT-qPCR assay have not been provided in the methods section. These details are vital to demonstrate efforts have been made to reduce false positive detection.

**Apologies we have corrected this error and included details on the inclusion of NTCs and referenced the previously generated *16S rRNA* standard curve providing a limit of detection** **^1^** **.   The raw qPCR data can also be found at** https://doi.org/10.17605/OSF.IO/V75DU.

1. Please clarify how Fisher’s tests were used to analyze your data. These tests were not mentioned in the methods section and are applied in pairwise comparisons, which might explain why some tests in Table 1 have such high P values.

**Apologies we erroneously missed a ‘statistics’ section in the methods and have now addressed this **

1. At the beginning of the results section it would be useful to include a brief summary of which mosquito species were collected, and where and when they were collected. The when is important given samples were collected across many years.

**Unfortunately there was a type for the collection ‘years’ for the Central region (this was only in 2019) and we have modified this in the methods.  This reduces any confusing in terms of ‘when’ they were collected and all location data is present in the methods so we don’t feel a summary is now warranted. **

1. I do not see an equivalent of table 2 for the central region data. It looks as though Table 2 just deals with the western region.

**That is correct.  Unfortunately we were unable to morphologically identify species in the Central region due to 1) a lack of expertise and 2)** **missing or damaged morphological features during mosquito collections.  Morphological identification of less-well known Culicine species is particularly challenging and often results in misidentification** **^2^** **. Therefore, we firstly** **screened individuals for symbionts using qPCR targeting both *Wolbachia* and *Asaia* *16S rRNA* genes to identify samples of interest (and limited our analysis to the genera level). MLST sequencing was then carried out on selected individuals for *Wolbachia* strain typing (our main aim of this work) followed by molecular confirmation of mosquito species using CO1 barcoding.  ** Queries about phylogenetic trees:

**-** In Fig. 3, is there an explanation for why Cx pipiens and Cx quinquefasciatus don’t cluster together. **There are multiple 16S sequences available on GenBank for *Wolbachia* strains detected in species within the *Culex pipiens*complex indicating genetic diversity in the strain group loosely labelled as ‘wPip’.  This would be reflective of numerous studies showing that there are multiple *Wolbachia* strains that co-existing within *Culex pipiens* populations** **^3-5^** **.  We have added a sentence to our discussion to highlight this point and the value of undertaking MLST to provide greater depth of sequencing data.** **- **None of the phylogenetic appear to have outgroups **Out grouping for both mosquito barcoding and Wolbachia phylogenetic trees, to our knowledge, is not commonly undertaken (at least based on the vast majority of similar publications).  Instead, we (and previously published studies) include mosquito barcoding gene sequences present on GenBank that closely match sequences generated in this study. For *Wolbachia* 16S, we (and previously published studies) include 16S sequences from well characterised Wolbachia strains such as wMel and wRi for context.** **- **Fig. 4 - while it is useful to have a tree based on the Wolbachia 16s gene. It could be valuable to include an unrooted consensus tree based on the MLST data. **Although we agree we were not able to generate complete MLST profiles for all mosquito species (please see table 5) which is consistent with numerous other published studies.  Therefore, we feel a phylogenetic tree based on MLST data would be incomplete and provide little beneficial data not present in table 5.  **

1. Is there a reason why Figure 2 and Table 2 collapse the prevalence of Wolbachia and Asaia to the genus level? This reduces the biological relevance of your findings as mosquitoes within a genus can fill distinct biological niches.

**I think this is referring to Figure 2 and yes we agree that it does limit our conclusions. Please see our response to point 5 but we have also included a few sentences in the discussion (paragraph 4) to highlight this.  **

1. Figure 2 is not visually accessible (red/green colors).

**Thank you for pointing this out – we have changed the colour scheme. **

1. The discussion describes the collection areas in Cameroon as containing a high degree of environment-driven variation. Reflecting on that, the decision to bin samples into two homogeneous regions (West vs Central) does not make sense. Are there any site-specific patterns that could be mentioned that better reflect site-to-site diversity in mosquito species/Wolbachia prevalence?

**We agree but unfortunately the collections were affected by the covid pandemic and we had to treat samples differently (individuals vs pools, morphological identification) between West and Central collections due to restricted travel and time working in labs.  In our methods we do include details on collection locations but do not feel we have sufficient ‘site-specific environmental data’ to justify further sub-divisions within the two Regions. **

1. The discussion described Wolbachia/Asaia co-infection as being “widespread” but this does not appear to reflect the data given that Asaia was only highly prevalent in one mosquito genus.

**We agree and have addressed this in our discussion (paragraph 4) and have changed the title to reflect this with a more balanced conclusion.  **

1. If the introduction were to briefly discuss the impact of Asaia on mosquito vector competence and immunity it could add important context outlining the rationale for studying Asaia infections in mosquitoes.

**We agree and have added some information on *Asaia* and its potential use in mosquito biocontrol strategies (please see paragraph 2).** **References**   1.         Walker, T., Quek, S., Jeffries, C.L., Bandibabone, J., Dhokiya, V., Bamou, R., Kristan, M., Messenger, L.A., Gidley, A., Hornett, E.A., et al. (2021). Stable high-density and maternally inherited Wolbachia infections in Anopheles moucheti and Anopheles demeilloni mosquitoes. Curr Biol *31*, 2310-2320 e2315. 10.1016/j.cub.2021.03.056. 2.         Jourdain, F., Picard, M., Sulesco, T., Haddad, N., Harrat, Z., Sawalha, S.S., Gunay, F., Kanani, K., Shaibi, T., Akhramenko, D., et al. (2018). Identification of mosquitoes (Diptera: Culicidae): an external quality assessment of medical entomology laboratories in the MediLabSecure Network. Parasit Vectors *11*, 553. 10.1186/s13071-018-3127-7. 3.         Morningstar, R.J., Hamer, G.L., Goldberg, T.L., Huang, S., Andreadis, T.G., and Walker, E.D. (2012). Diversity of Wolbachia pipientis strain wPip in a genetically admixtured, above-ground Culex pipiens (Diptera: Culicidae) population: association with form molestus ancestry and host selection patterns. J Med Entomol *49*, 474-481. 4.         Atyame, C.M., Delsuc, F., Pasteur, N., Weill, M., and Duron, O. (2011). Diversification of Wolbachia endosymbiont in the Culex pipiens mosquito. Mol Biol Evol *28*, 2761-2772. 10.1093/molbev/msr083. 5.         Duron, O., Raymond, M., and Weill, M. (2011). Many compatible Wolbachia strains coexist within natural populations of Culex pipiens mosquito. Heredity (Edinb) *106*, 986-993. 10.1038/hdy.2010.146.
